# How accurate is the diagnosis of rheumatic fever in Egypt? Data from the national rheumatic heart disease prevention and control program (2006-2018)

**DOI:** 10.1371/journal.pntd.0008558

**Published:** 2020-08-17

**Authors:** Alaa Ghamrawy, Nermeen N. Ibrahim, Ekram W. Abd El-Wahab

**Affiliations:** 1 Department of Non-Communicable Diseases, Ministry of Health and Population, Cairo, Egypt; 2 Department of Epidemiology, High Institute of Public Health, Alexandria University, Alexandria, Egypt; 3 Department of Tropical Health, High Institute of Public Health, Alexandria University, Alexandria, Egypt; Yale University School of Medicine, UNITED STATES

## Abstract

Rheumatic heart disease (RHD) as a chronic sequela of repeated episodes of acute rheumatic fever (ARF), remains a cause of cardiac morbidity in Egypt although it is given full attention through a national RHD prevention and control program. The present report reviews our experience with subjects presenting with ARF or its sequelae in a single RHD centre and describes the disease pattern over the last decade. A cross-sectional study was conducted in El-Mahalla RHD centre between 2006 and 2018. A total of 17014 individual were enrolled and evaluated. Diagnosis ARF was based on the 2015 revised Jones criteria and RHD was ruled in by echocardiography. The majority of the screened subjects were female (63.2%), in the age group 5–15 years (64.6%), rural residents (61.2%), had primary education (43.0%), and of low socioeconomic standard (50.2%). The total percentage of cases presenting with ARF sequelae was 29.3% [carditis/RHD (10.8%), rheumatic arthritis (Rh.A) (14.9%), and Sydenham’s chorea (0.05%)]. Noticeably, 72% were free of any cardiac insult, of which 37.7% were victims of misdiagnoses made elsewhere by untrained practitioners who prescribed for them long term injectable long-acting penicillin [Benzathine Penicillin G (BPG)] without need. About 54% of the study cohort reported the occurrence of recurrent attacks of tonsillitis of which 65.2% underwent tonsillectomy. Among those who experienced tonsillectomy and/or received BPG in the past, 14.5% and 22.3% respectively had eventually developed RHD. Screening of family members of some RHD cases who needed cardiac surgery revealed 20.7% with undiagnosed ARF sequalae [RHD (56.0%) and Rh.A (52.2%)]. Upon the follow-up of RHD cases, 1.2% had improved, 98.4% were stable and 0.4% had their heart condition deteriorated. Misdiagnosis of ARF or its sequelae and poor compliance with BPG use may affect efforts being exerted to curtail the disease. Updating national guidelines, capacity building, and reliance on appropriate investigations should be emphasized. Since the genetic basis of RHD is literally confirmed, a family history of RHD warrants screening of all family members for early detection of the disease.

## Author summary

Rheumatic heart disease (RHD), a chronic sequela of acute rheumatic fever (ARF), remains the leading cause of acquired paediatric heart disease, premature death and disability in indigenous Egyptian populations. The disease affects mainly children and young adults in their most productive years, adding to the economic burden of the nation. The WHO and the Egyptian Ministry of Health and Population have jointly launched a national RHD prevention and control program in 2006, to tackle ARF and RHD at the local level. The comprehensive program provides decentralized primordial, primary, secondary and tertiary care services through 30 regional satellite centres that have been set up around the country. We analysed data of 17050 subjects evaluated through the program in El-Mahalla RHD centre over 12 years (2006–2018). The results revealed that ARF is often associated with cardiac affection and shares a similar set of risk factors in the population. However, the percentage of newly detected rheumatic cases is falling overtime albeit with increasing numbers of ARF/RHD misdiagnoses. The program is scaling-up its activities and is giving way to achieve its goals through improving social standards, intensifying community awareness, capacity building and the provision of better health care for all.

## Introduction

Acute rheumatic fever (ARF) is a multisystemic sequela of a post infection autoimmune reaction due to untreated group A β haemolytic *Streptococcus* (GABHS) pharyngitis and possibly pyoderma [1,2]. The main features of ARF include elevated inflammatory and GABHS serological markers, as well as one or more of carditis, rheumatic arthritis (Rh.A), skin/soft tissue manifestations or chorea. Recurrent episodes of ARF and its associated cardiac inflammation ultimately lead to permanent valvular damage and rheumatic heart disease (RHD) [2]. Molecular mimicry between the GABHS and heart or brain structures underlays the immune responses in ARF, where cross-reactive antibodies and T-cells that react with self-antigens are the effectors of valve or brain tissue damage [1,3,4]. The risk of ARF recurrences increases following the initial episode, although it can be largely prevented by secondary prophylaxis with injectable long-acting penicillin [benzathine penicillin G (BPG] every 3–4 weeks [5,6].

ARF and its sequel, RHD are serious but preventable health conditions that can occur in children and young adults. Except for a lingering burden in elderly people, ARF and RHD are virtually disappearing in high-income countries due to improved living conditions and medical care. However, these conditions are not receiving enough attention and are inadequately controlled in most of the low resource countries [3,7]. Apparently, demographic shifts and immigration from low-income to high-income settings might be responsible for a new burden of RHD in regions in which the disease has been eliminated [8]. The disease is prevalent in developing regions that are characterized by impoverished socioeconomic determinants [3,7]. Of concern, the Middle East, the Indian subcontinent, and some areas of Africa and South America, that show over 20 million new cases each year. Globally, 30 million people are currently affected by RHD, with 233,000 directly attributable deaths. This represents 25–40% of all cardiovascular disease and accounts for 25–50% of all cardiac admissions [9].

The pathogenesis of ARF remains enigmatic, and specific treatment is not available. Yet, the prevention of the initial and recurrent attacks is possible with penicillin treatment. In Egypt, the prevalence of RHD has been declining over the past 3 decades and has currently plateaued at ~10/1000 population [10,11]. There are actually 300,000 patients with RHD aged 5 to 15 years of age [12]. These patients require adequate medical care to prevent their progression to severe valvular damage and save their lives. If these patients are neglected, they will require 300,000 open-heart surgeries, which will cost the nation 1.7 billion US$ dollars.

### The National RHD prevention and control program in egypt

**Fig 1:**
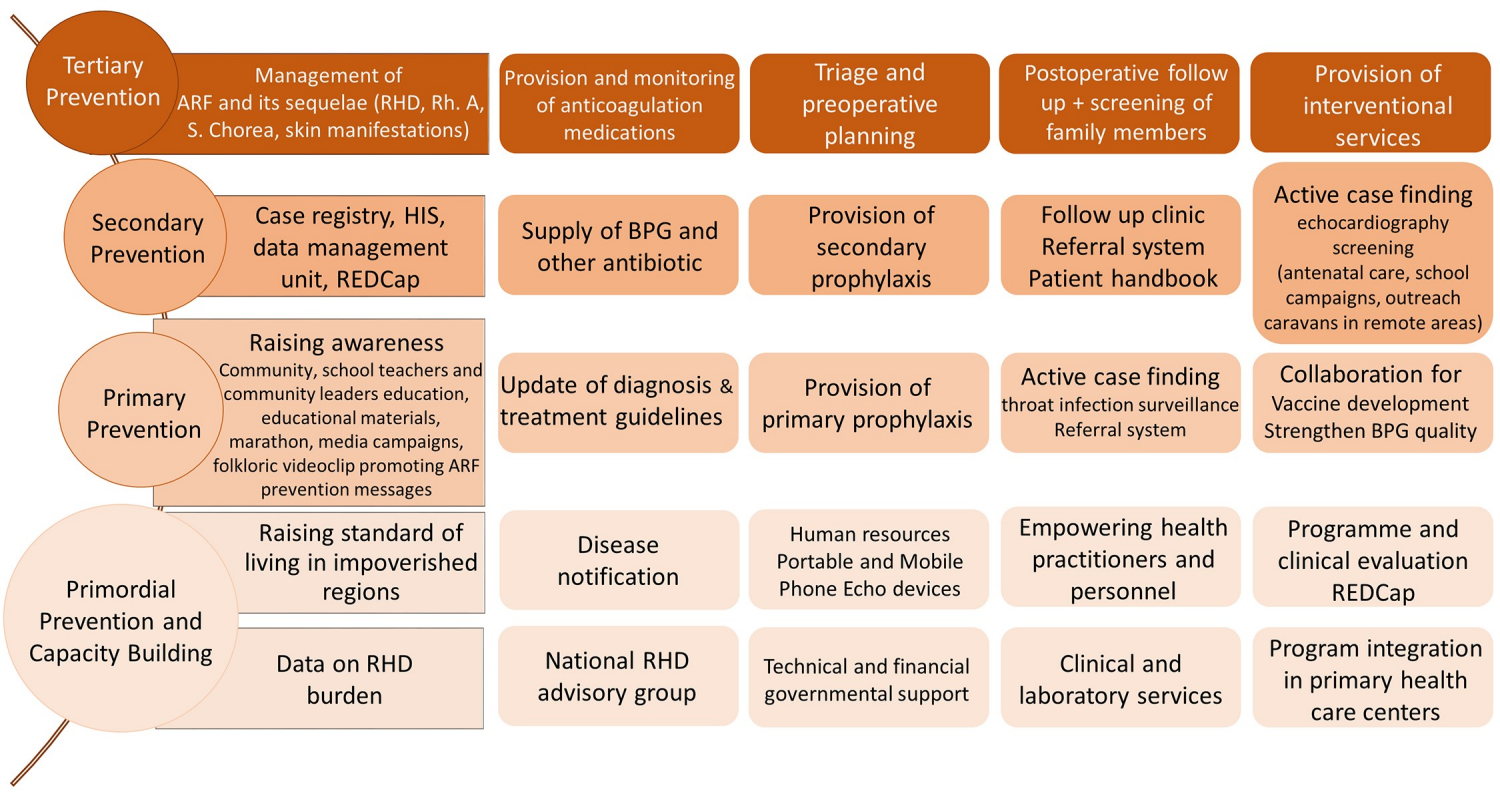
Components of the national rheumatic heart disease prevention and control program founded in Egypt in 2006. The program follows the conceptual framework for comprehensive RHD control programs. **Footnote of Fig 1:** ARF; acute rheumatic fever, BPG; benzathine penicillin G, HIS; Health Information Systems, REDcap; Research Electronic Data Capture, Rh.A; rheumatic arthritis, RHD; rheumatic heart disease, S. Chorea; Sydenham’s Chorea.

RHD is a national issue in Egypt and is being given priority by the Egyptian Ministry of Health and Population (MOHP) under the auspices of all the experts and leaders concerned. The national RHD prevention and control program was established in 2006 through the RHD centre in Mahalla El-Koubra province, in collaboration with the WHO-Egypt. It was expanded in 2010–2014 to include 30 RHD primary care centres covering the country and are linked to tertiary-level of care cardiac centres. The ultimate goal is to reduce mortality from RHD and declare Egypt free of ARF by 2030. The program conforms to the international guidelines for the identification and management of pharyngitis, ARF and RHD, which are approached from a variety of angles [6,8,13,14,15]. Guided by the conceptual framework for comprehensive RHD control programs developed by Wyber [6,16], the program structure falls into primordial, primary, secondary and tertiary levels of prevention that prioritize capacity building, raising awareness as well as disease surveillance and management (Fig 1).

The program involves the identification of ARF and RHD among the suspected cases referred to RHD centres. There is no age limit for the screening and the screening materials target primarily at RHD and ARF. For instance, school-age children are screened at school entry by trained physicians using auscultation. Suspected cases are then referred to specialized RHD centres for confirmation by echocardiography. Since mild RHD is asymptomatic, and patients with RHD do not seek health care except at late stages of the disease or when complications develop, the program implements active case identification through large-scale field echocardiography screening campaigns and outreach caravans (community- and school-based) using portable and mobile echocardiography. In addition, screening of pregnant women is performed in antenatal care. As a part of post-operative planning for RHD patients who underwent cardiac surgery, their family members are invited to screening for RHD.

Other components of the program include case registry for the registration of identified rheumatic cases, raising awareness for management of GABHS infection, ARF and RHD through mass media and campaigns which target health care workers, teachers, youth centres, civil societies, clergy, community leaders, and the public at large. A video clip of a folkloric song in local language for children was developed for promoting health message for ARF and RHD prevention. The song was created by the authors and recorded by a famous Egyptian folklore singer to be disseminated through the different media channels and in the daily health education sessions held by the program (S1 Video with English subtitles).

Because the program is run via the Egyptian MOHP, an emphasis is placed on capacity building, both in public health and medical sectors. Hundreds of health care workers are trained to recognize the clinical signs and symptoms of GABHS pharyngitis and to administer proper treatment with one-time BPG injection (S1 Table). Training materials, including clinical guidelines [15], are distributed to health professionals down to the health unit level in which providers are instructed to refer patients with symptoms for formal evaluation with echocardiography at the specialized regional centres. Practical actions are being taken to improve the standard of living in impoverished and disadvantaged regions. Work to strengthen the quality of BPG and collaborative research with Sir. Magdy Jacob Research Centre (United Kingdom) to develop a vaccine for GABHS are underway.

We share in this local experience the epidemiological features of ARF and its sequelae a population with very high disease rates, how accurate was their diagnosis made by health practitioners, and the compliance with the use of BPG, in El–Mahalla RHD centre where the national RHD prevention and control program was first established.

## Methods

### Ethics statement

The study was approved by the institutional review board and the ethics committee of the High Institute of Public Health affiliated with Alexandria University, Egypt (ref: 305–2018). The study was conducted in accordance with the international ethical guidelines and of the Declaration of Helsinki [103]. Informed written consent was obtained from each participant after explaining the aim and concerns of the study. Parents, guardians and caregivers provided written consent on behalf of children and those with mental disabilities. Data sheets were coded by number to ensure anonymity and confidentiality of the participants’ data.

### Study setting and design

A cross-sectional study was conducted through the national RHD prevention and control program implemented in El–Mahalla RHD centre. The RHD centre in El–Mahalla was the first established centre to run the RHD program in 2006, owing to the high burden of the ARF/RHD cases identified and refereed there. Starting from 2010, several centres were established in other cities in Egypt until reaching 30 centres in 2014. Unlike the other centres, the program data in El–Mahalla RHD centre covers longer a period of time dating to 2006. The centre is located in Mahalla El-Koubra province, which is one of eight administrative districts of the Gharbia governorate (Northern Egypt) with ~2 million inhabitants [17].

### Study period

The study was conducted between 2006 and 2018. Data about the economic burden of RHD at El-Mahalla cardiac centre covered the period 1998–2018.

### Study population

Over the last decade (2006–2018), a total of 17014 suspected ARF and RHD patients were referred to the RHD centre in the El-Mahalla. Those referred for consideration comprised three groups:

i) anyone with symptoms suggesting ARF/RHD who sought medical advice at El-Mahalla RHD centre after learning about the program from relatives, neighbours, advertisement, media or the program folkloric song, ii) those with known RHD condition as has been tentatively diagnosed in other health care settings and referred to El-Mahalla RHD centre. Interestingly, a large number of this particular group were misguidedly prescribed long term BPG for secondary prophylaxis, without being confirmed as having RHD by echocardiography. Instead, their health care physicians were relying on symptoms suggesting RHD [breathlessness, dyspnoea on effort in patients with a history of recurrent attacks of sore throat/tonsillitis, joint pain, arthritis] and the elevated ESR for establishing the diagnosis of RHD. We refer thereafter to this group of patients as “misdiagnoses”, iii) school children screened at school entry by school physician. Those suspected to have ARF or RHD are referred to El-Mahalla RHD centre, iv) family members of patients with established RHD who had cardiac surgery. These included a total of 259 RHD patients who were primarily screened through the RHD program in El-Mahalla centre and eventually underwent cardiac surgery for RHD. Their valvular condition was severe and indicated the need for valve replacement cardiac surgery which was done at a tertiary care cardiac centre. After being operated, we invited them to bring their children/household members to check for RHD. The total number of the screened relatives of these 259 RHD patients was 769 family members.

All referred subjects were enrolled in the program and evaluated accordingly. Although the program involves case registry and follow-up of the identified ARF and RHD patients as being undertaken at all RHD centres in Egypt, this report is confined to one-time information gathered at the screening phase. One protocol was applied to all subjects referred to the centre.

### Screening and identification of ARF and RHD cases

Screening of enrolled subjects included history taking, clinical evaluation through general physical examination, auscultation, ECG and echocardiography, and case review by a cardiologist.

Data collection was done through a data collection sheet which included two sections: a) a pre-designed structured interview questionnaire that covered baseline sociodemographic data, history of ARF manifestations and its sequelae, and the use of BPG. According to El-Gilany et al. [18], the socioeconomic status was determined based on the level of education, occupation, income per capita, residency, crowding index (the total number of co-residents per household, excluding the new-born infant, divided by the total number of rooms, excluding the kitchen and bathrooms) [19], and sanitation (presence of safe water supply, electricity, household water closet, sewage disposal, and garbage disposal). Non-adherence to BPG use for secondary prophylaxis for all individuals who have had an initial attack of ARF [20] was defined as patients taking <80% of the prescribed medication [21]; b) a sheet for the presenting clinical data of each individual including auscultation, ECG and the results of the Doppler echocardiography which is considered the most important criterion for the diagnosis of RHD. For the case diagnosis of ARF, the revised Jones criteria [15] were applied. Arthritis was diagnosed by the presence of swelling, pain, heat in the joints, and limitations in joint movement. Atypical joint involvements [mono- or oligoarticular, additive, symmetric, involving small joints of the hands and feet, and persistent] [22] were also considered to avoid under diagnosis. The diagnosis of Sydenham’s chorea was based on the exclusion of other causes and forms of chorea. In some cases, Sydenham’s chorea was found in presence of arthritis and/or carditis. Normal peers were defined as screened individuals who were found free of any rheumatic condition (those who were referred and were not diagnosed with ARF or its complications, normal family members of cardiac surgery patients who were screened and found free of ARF or its sequelae, and individuals with prior misdiagnoses).

### Laboratory investigations

Indicators of inflammation including the erythrocyte sedimentation rate (ESR), Antistreptolysin-O titre (ASOT), and C-reactive protein (CRP) were done only for acute cases, whether cases of carditis or arthritis. A value above 20 mmHg/first hour for ESR was considered elevated. ASOT greater than 200 IU was considered abnormal and an evidence of a recent GABS infection [7]. No serial ASOT measurements were performed because it was not feasible to obtain acute and convalescent sera. Most of the patients were presenting within a relatively late phase of the disease or with delayed manifestations of ARF where ASOT is already stabilized. Moreover, a rise in ASOT is less prominent in recurrent ARF compared to 1st attack, and acute and recurrent tonsillitis [23]. It therefore loses its value as an index of the inflammatory activity of the disease [24], although an absolute value of ASO titre can be of diagnostic importance [25].

To confirm the diagnosis of RHD, each individual underwent ECG and colour Doppler echocardiography. For a definite diagnosis of RHD, detailed transthoracic two-dimensional and Doppler studies were performed in all cases by experienced echocardiographers and reviewed by consultant cardiologists for confirming the diagnosis. Echocardiographic diagnosis of RHD was performed according to the standard criteria set by the 2012 World Heart Federation [8], using GE. RT 6800 and Siemens Acuson CV-70 ultrasound systems. Briefly, standard transthoracic echocardiography (GE, Vivid 8, Chicago, USA) was performed in which 2D, M-mode, colour and Doppler modalities were used with images taken from the parasternal long axis, parasternal short axis, apical four chamber, apical five chamber and subcostal views. The combined use of Doppler-based and morphology-based criteria (any amount of valvular regurgitation (regurgitant jet >1 cm in length, regurgitant jet in at least two planes, mosaic colour jet with a peak velocity >2.5 m/sec, and persistent jet throughout systole or diastole) noted in at least two planes associated with at least two of the following morphological signs: restricted leaflet mobility, abnormal subvalvular thickening, or focal or generalized valvular thickening) had a three to four times higher rate of detection of subclinical RHD compared with the exclusive use of Doppler-based criteria [26]. The severity of valvular lesion was qualitatively graded from 0 to 4 (0: absent, 1; trivial, 2; mild, 3; moderate, 4; severe) [14,27,28].

Detected cases were offered a free standard of care according to their health status [29]. Antibiotic prophylaxis as well as echocardiography check-ups were offered at no expense to all patients, while those in need of heart surgery were referred to El-Mahalla cardiac centre (a tertiary care centre which provides tertiary care for RHD patients referred from El-Mahalla RHD centre as well as for congenital heart disease and coronary artery disease cases refereed from other primary health care centres).

### Prevention of ARF and RHD

Antibiotic treatment of GABHS infections to prevent first attacks of ARF (primary prophylaxis) or prophylaxis for preventing its recurrent episodes (secondary prophylaxis) are given according to the regimens described in S1 Table [30].

### Statistical analysis

The collected data were reviewed for accuracy and integrity and fed into computer software. Data were analysed using a statistical software package (SPSS Statistics Base 21.0, IBM, Armonk, NY). Continuous variables were presented as the mean ± standard deviation (SD). Categorical variables were expressed as numbers with proportions, n (%). Variables relevant to laboratory data were dichotomized according to prefixed cut-offs, taking into consideration the normal reference values.

We compared the baseline sociodemographic, clinical, and laboratory characteristics of individuals with ARF/RHD or individuals with misdiagnoses with completely free subjects. Continuous variables were compared using an independent-samples *t*-test. Categorical variables were compared by chi-squared test or Fisher’s exact test. The variables associated with ARF/RHD at the *p* <0.05 significance level in univariate analyses and deemed potentially useful for clinical prediction, were selected for multivariable analysis. The selected variables were entered as covariates to develop a multivariable logistic regression equation by backward Wald stepwise elimination, with rheumatic condition/misdiagnosis being the outcome variable.

## Results

### Burden of RHD from 1998 to 2018

**Fig 2:**
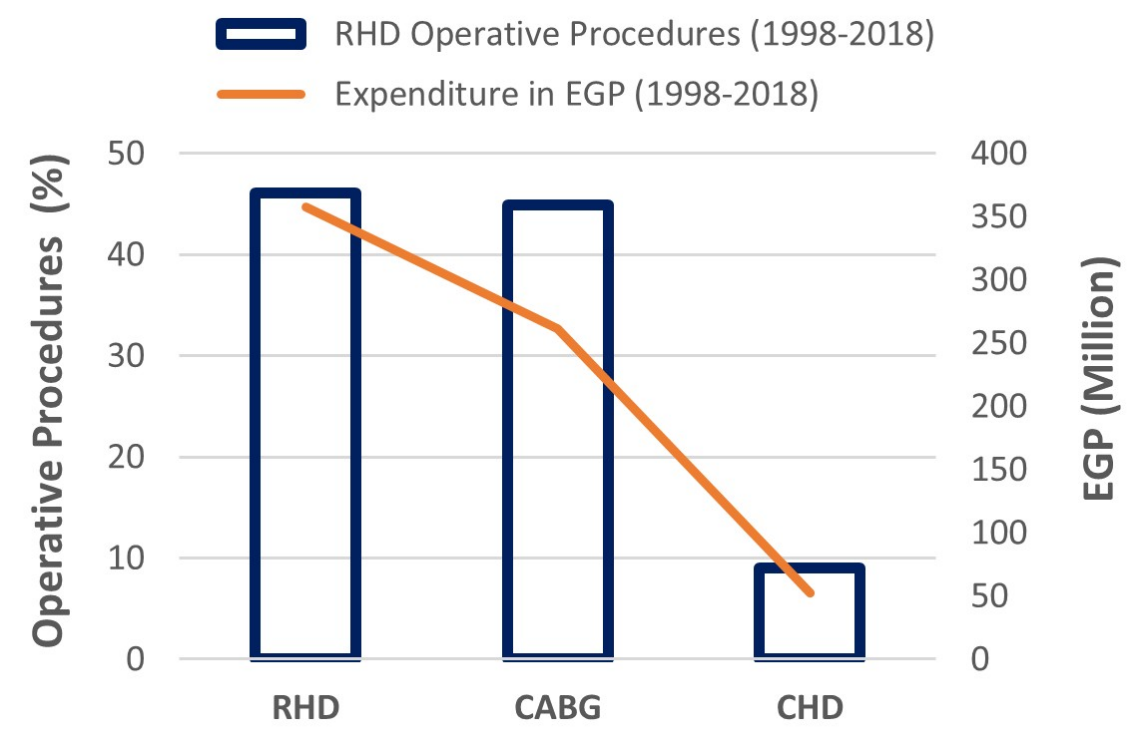
Total number of RHD operative procedures and expenditures in the El-Mahalla tertiary care cardiac centre (1998–2018). **Footnote of Fig 2:** RHD; rheumatic Heart disease, CABG; Coronary artery bypass grafting, CHD; Congenital heart disease, EGP; Egyptian pound.

Between 1998–2018, El-Mahalla cardiac centre conducted 2680 open-heart surgeries for valve replacement in complicated RHD cases referred from El-Mahalla RHD centre. This represented 46.1% of all cardiac operative procedures performed in El-Mahalla cardiac centre alone, and placed a total cost of approximately 358 million Egyptian pounds (EGP) (Fig 2).

**Fig 3:**
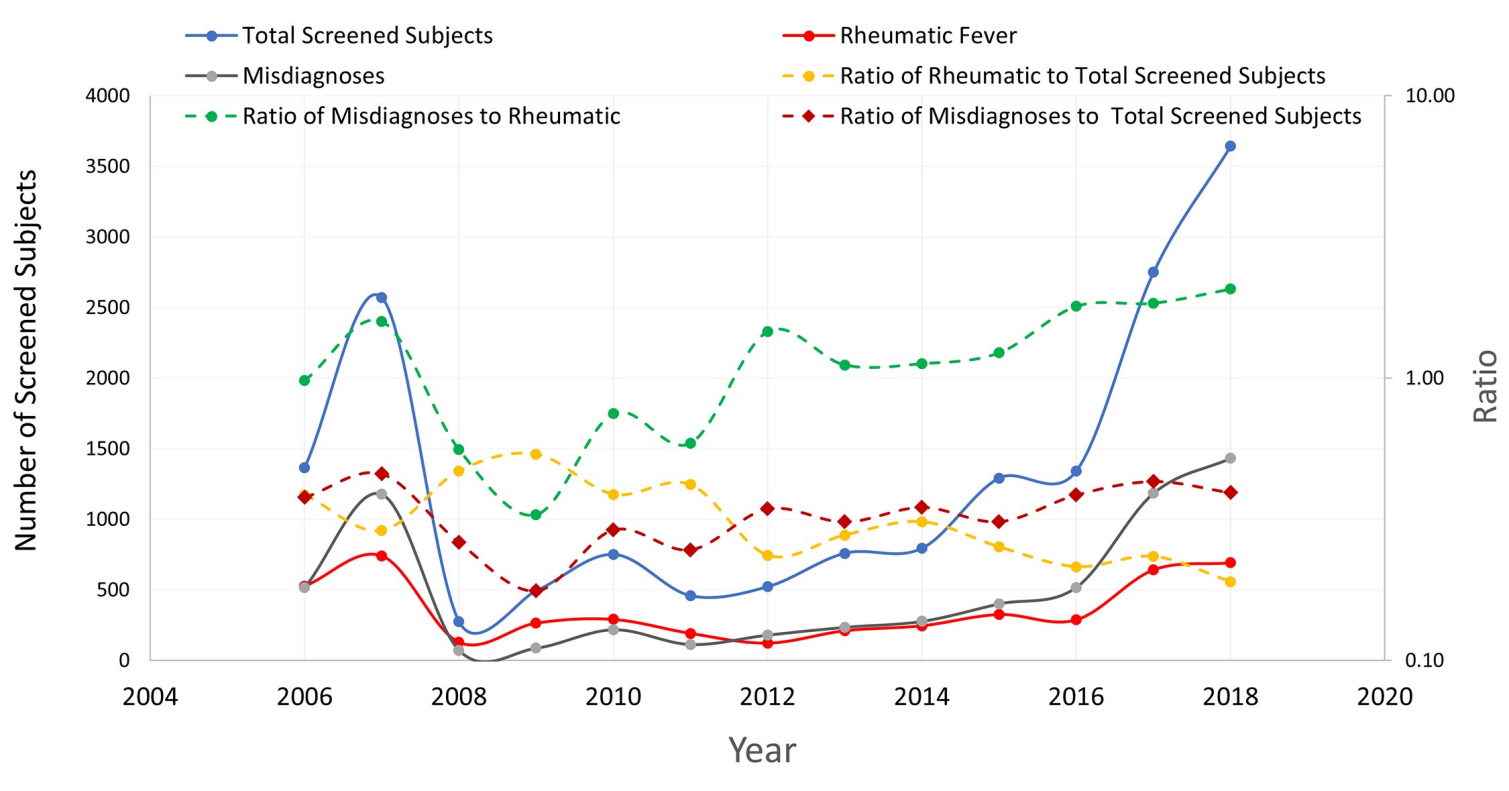
Number of screened subjects versus the number of cases diagnosed with rheumatic fever or its sequelae per year, in El-Mahalla canter (2006–2018).

During the study period (2006–2018), and among a total of 17014 suspected rheumatic cases, an average of 1308 subjects were screened per year. We identified an average of 360 new cases with ARF/RHD per year [average 32.3% (±10.7) of the screened subjects]. The number of screened subjects rose steeply late in the decade. Meanwhile, the proportion of the identified rheumatic patients showed a continuous decline over time, whereas that of misdiagnoses lingered behind (Fig 3). The number of cases diagnosed with Rh.A was consistently higher compared to the number of confirmed RHD cases (average 164 cases per year), although the latter showed an unusual peak in 2018 (S1 Fig).

The highest disease incidence season was late in winter and early in spring (February to April, with 27.8% of cases), closely followed by summer and early autumn (July to September, with 25.0% of cases) (S2 Fig).

### Characteristics of the screened cohort

The overall mean age of the evaluated cohort was 13.5 ± 8.5 years (age range 2–69, median = 11). The majority were in the age group 5–15 years (64.6%), female (63.2%), rural residents (61.2%), single (52.5%), educated at the primary level (43.0%), and of low socioeconomic status (50.2%). Approximately 2.4% were smokers [85.3% in age group >25 years and 14.7% in age group (18 - <25 years)], whereas 27.5% experienced second-hand smoking [86.3% in children <18 years, 10.3% in youth (18 - <25 years) and 3.4% in adults > 25 years]. Most of the screened individuals (87.3%) were referred to the centre by health care providers, although 23.7% reported having no access to health care services (Table 1).

**Table 1:** Sociodemographic characteristics of the screened cohort.

	Total Participants (n = 17014)				Total		Univariate analysis	Multivariate analysis			
	Normal (n = 12348)		Rheumatic (n = 4666)					Exp(B)	95% C.I. for EXP(B)		*p*
	n	%	n	%	n	%	*p*		LL	UL	
**Age (Years)**											
<5	654	5.3	95	2.0	749	4.4					**0.008**
5 –<15	8289	67.1	2706	58.0	10995	64.6		1.03	0.72	1.48	0.867
15 –<30	2705	21.9	1398	30.0	4103	24.1	**<0.001**	**1.87**	**1.11**	**3.16**	**0.019**
30–50	678	5.5	411	8.8	1089	6.4		1.93	0.93	4.02	0.079
50+	22	0.2	56	1.2	78	0.5		**5.62**	**1.80**	**17.6**	**0.003**
**(Mean ± SD)**	12.7 ± 7.9		15.6 ± 9.8		13.5 ± 8.5		**<0.001**				
**Smoking**											
Yes	259	2.1	149	3.2	408	2.4	**<0.001**	1.29	.961	1.72	0.090
Passive (second hand smoking)	3659	29.6	1029	22.1	4688	27.5	0.08	**1.14**	**1.02**	**1.27**	**0.016**
**Sex**											
Male	4795	38.8	1464	31.4	6259	36.8	**<0.001**				
Female	7553	61.2	3202	68.6	10755	63.2		**1.20**	**1.10**	**1.32**	**<0.001**
**Residence**							** **				
Rural	7755	62.8	2661	57.0	10416	61.2	**<0.001**				
Urban	4593	37.2	2005	43.0	6598	38.8		**1.16**	**1.06**	**1.27**	**0.001**
**Education**							** **				
Illiterate	237	1.9	167	3.6	404	2.4	**<0.001**				0.053
Read and Write	297	2.4	252	5.4	549	3.2		1.53	0.80	2.92	0.195
Kindergarten	1874	15.2	333	7.1	2207	13.0		**1.89**	**1.08**	**3.29**	**0.025**
Primary	5485	44.4	1835	39.3	7320	43.0		**1.32**	**1.05**	**1.66**	**0.017**
Preparatory	1921	15.6	821	17.6	2742	16.1		**1.46**	**1.10**	**1.94**	**0.009**
Secondary	1921	15.6	961	20.6	2882	16.9		1.24	0.78	1.98	0.354
University	613	5.0	297	6.4	910	5.3		1.02	0.56	1.87	0.950
**Father's occupation**											
Working	11159	94.9	3997	92.1	15156	94.3	**<0.001**				
Not working	244	2.1	118	2.7	362	2.3					
Retired	354	3.0	223	5.1	577	3.6					
**Mother's occupation**							** **				
Working	1835	15.0	564	12.3	2399	14.1	** <0.001**				
Not Working	10391	85.0	4025	87.7	14416	84.7		**1.26**	**1.14**	**1.39**	**.004**
**Parents**											
Alive	11744	95.2	4323	92.5	16067	94.4	**<0.001**				
Died	597	4.8	350	7.5	947	5.6					
**Marital status**							** **				
Single	1565	54.6	766	48.7	2331	52.5	**<0.001**	**0.66**	**0.55**	**0.80**	**<0.001**
Married	1267	44.2	791	50.3	2058	46.4		0.91	0.73	1.14	0.404
Widowed	11	0.4	7	0.4	18	0.4		0.33	0.10	1.02	0.054
Divorced	23	0.8	9	0.6	32	0.7		0.93	0.38	2.25	0.869
**Income**											
Not enough	6125	49.6	2411	51.6	8536	50.2	**0.022**	**1.57**	**1.43**	**1.72**	**<0.001**
Enough	6216	50.4	2262	48.4	8478	49.8					
**Family Size**							** **				
1–3	743	6.0	283	6.1	1026	6.0					
4–8	11494	93.1	4300	92.2	15794	92.8	**<0.001**				
>8 (9–15)	111	0.9	83	1.8	194	1.1					
**(Mean ± SD)**	12.7 ± 7.9		15.6 ± 9.8		13.5 ± 8.5		**<0.001**	**1.05**	**1.01**	**1.1**	**0.014**
**Crowding Index**							** **				
1–2	11960	96.9	4391	94.1	16351	96.1					0.009
3–5	342	2.8	242	5.2	584	3.4	**<0.001**	**1.55**	**1.13**	**2.13**	**0.007**
>5 (6–11)	46	0.4	33	0.7	79	0.5		**3.02**	**1.35**	**6.78**	**0.007**
**(Mean ± SD)**	1.6 ± 0.62		1.7 ± 0.8		13.5 ± 8.5		**<0.001**	** **	** **	** **	** **
**Ventilation**							** **				
Good	9003	72.9	2823	60.5	11826	69.5					**<0.001**
Average	3071	24.9	1619	34.7	4690	27.6	**<0.001**	**1.72**	**1.54**	**1.91**	**<0.001**
Bad	274	2.2	224	4.8	498	2.9		**1.75**	**1.31**	**2.34**	**<0.001**
**Medical service**							** **				
None	2443	19.8	1591	34.0	4034	23.7	**<0.001**	**1.85**	**1.17**	**2.93**	**0.009**
Private health care services	6814	55.2	2311	49.5	9125	53.6		1.18	0.74	1.90	0.482
Health Insurance	2259	18.3	545	11.7	2804	16.5		0.86	0.54	1.38	0.542
PHC units	535	4.3	84	1.8	619	3.6		0.74	0.43	1.29	0.285
University Hospitals	8	0.1	3	0.1	11	0.1		1.40	0.13	15.12	0.781
Public Hospitals	282	2.3	139	3.0	421	2.5		** **	** **	** **	**<0.001**
**Referral method**											
Health care Physician	10274	83.3	4584	98.1	14858	87.3	**<0.001**	**6.98**	**2.56**	**19.07**	**<0.001**
Family	638	5.2	18	0.4	656	3.9		0.91	0.30	2.77	0.862
Advertisement	928	7.5	59	1.3	987	5.8		1.39	0.49	3.94	0.540
Friend	3	0.0	1	0.0	4	0.0		3.76	0.22	65.4	0.364
Neighbours	426	3.5	5	0.1	431	2.5		0.29	0.07	1.10	0.068
Others	72	0.6	6	0.1	78	0.5					**<0.001**

PHC = primary health care

### Characteristics of rheumatic patients

**Fig 4:**
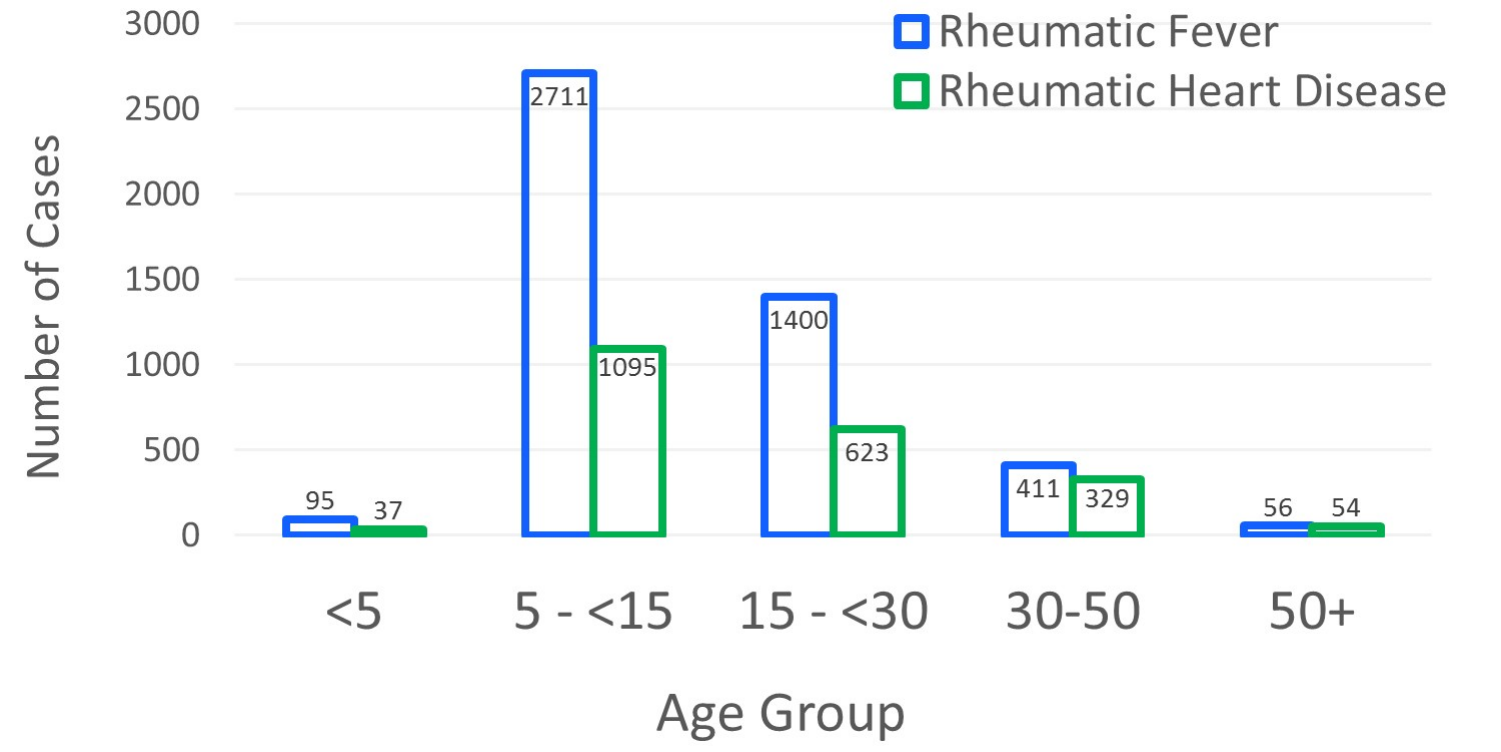
Age distribution of acute rheumatic fever and rheumatic heart disease patients.

Among the screened individuals attending El-Mahalla RHD centre, a total of 4666 (27.4%) subjects were rheumatic presenting with ARF or its sequelae [carditis/RHD (10.8%), Rh.A (14.9%), Sydenham’s chorea [a total of 8 cases (0.05%); combined with carditis/RHD in 6 cases (75.0%)], and both carditis/RHD and Rh.A (1.8%)]. Importantly, the disease was ruled out in 37.7% of the enrolled subjects (misdiagnoses) who were referred to the centre as ARF/RHD cases. The predominant sociodemographic and clinical features of rheumatic patients are depicted in Table 1, Table 2 and Fig 4.

**Table 2:** Clinical presentation of the screened cohort.

	Total Participants (n = 17014)				Total		Univariate analysis	Multivariate analysis			
	Normal (n = 12348)		Rheumatic (n = 4666)					Exp(B)	95% C.I. for EXP(B)		*p*
	n	%	n	%	n	%	*p*		LL	UL	
**Symptoms**											
Tonsillitis	3101	25.1	1564	33.5	4665	27.4	**<0.001**	**1.31**	**1.20**	**1.42**	**<0.001**
Pharyngitis	995	8.1	427	9.2	1422	8.4	**0.024**	1.10	0.98	1.30	0.081
Breathlessness/Dyspnea on effort	7278	58.9	2043	43.8	9321	54.8	**0.004**	**0.78**	**0.71**	**0.86**	**<0.001**
Arthralgia	932	7.5	42	0.9	974	5.7	**<0.001**	**0.21**	**0.15**	**0.29**	**<0.001**
Arthritis	623	5.0	1935	41.5	2558	15.0	**<0.001**	**11.0**	**9.70**	**12.4**	**<0.001**
Fever	279	2.3	182	3.9	461	2.7	**<0.001**	1.05	0.83	1.31	0.696
Others	125	1.0	18	0.4	143	0.8	**<0.001**	**0.22**	**0.12**	**0.42**	**<0.001**
**Recurrent attacks of tonsillitis**											
Yes	5803	47.0	2769	59.3	8572	50.4	**<0.001**				**<0.001**
≤ 6 attacks per year	5121	41.5	2354	50.4	7475	43.9	**<0.001**	**1.70**	**1.20**	**2.40**	**0.001**
> 6 attacks per year (up to 27)	682	5.5	415	8.9	1097	6.4		**2.80**	**2.00**	**4.00**	**<0.001**
**Tonsillectomy**											
Yes	3831	31.0	1755	37.6	5586	32.8	**<0.001**				
**Hospitalization**											
Cardiac	40	0.3	114	2.4	154	0.9	**<0.001**	**2.80**	**1.80**	**4.40**	**<0.001**
Others	805	6.5	438	9.4	1243	7.3	**<0.001**	1.10	0.90	1.20	0.410
**Echocardiography**											
Normal	12348	100.0	2535	54.3	14876	87.4					
MS	0	0.0	25	0.5	25	0.1					
MR	0	0.0	1740	37.3	1740	10.2					
AS	0	0.0	6	0.1	6	0.0					
AR	0	0.0	90	1.9	90	0.5					
TR	0	0.0	233	5.0	233	1.4					
AR + MR	0	0.0	31	0.7	31	0.2					
MR + MS + AR	0	0.0	4	0.1	4	0.0					
MS + AR	0	0.0	3	0.1	3	0.0					
MR + TR	0	0.0	1	0.0	1	0.0					
MR + MS	0	0.0	4	0.1	4	0.0					
MR + AS + AR	0	0.0	1	0.0	1	0.0					
**Degree of valvular affection**											
Trivial	0	0.0	241	11.3	241	1.4					
Mild	0	0.0	1635	76.5	1635	9.6					
Moderate	0	0.0	220	10.3	220	1.3					
Sever	0	0.0	42	1.9	41	0.2					
**Diagnosis**											
Normal	5930	48.1	0	0.0	5930	34.8					
Others (misdiagnosis)	6411	51.9	0	0.0	6411	37.7					
Carditis/RHD	0	0.0	1830	39.2	1830	10.8					
Rheumatic arthritis	0	0.0	2533	54.2	2533	14.9					
Sydenham’s chorea	0	0.0	2	0.0003	2	0.0					
RHD and Rheumatic arthritis	0	0.0	302	6.5	302	1.8					
RHD and Sydenham’s chorea	0	0.0	6	0.1	6	0.0					
**Major criteria of RF**											
Carditis	0	0.0	2138	45.8	2138	12.6					
Arthritis	0	0.0	2835	60.7	2835	16.7					
Sydenham’s Chorea	0	0.0	8	0.2	8	0.0					
**Minor criteria of RF**											
ASOT (Mean ± SD)	424.2 ± 167.6		438.0 ± 181.0		427.4 ± 170.4		0.460				
ESR 1st hr (Mean ± SD)	20.7 ± 13.3		26.4 ± 17.3		96.1 ± 22.3		**0.002**				
**History of receiving BPG**							**<0.001**				
Yes	6430	52.1	3406	72.9	9836	57.8		**3.30**	**2.90**	**3.80**	**<0.001**
**BPG regimen**							** **				
2 weeks	5611	87.3	2853	83.8	8464	86.1	**<0.001**				
3 weeks	158	2.5	96	2.8	254	2.6					
4 weeks	661	10.3	457	13.4	1118	11.4					
Adherent	4039	62.8	1820	53.4	5859	59.6	**<0.001**	**0.75**	**0.67**	**0.84**	**<0.001**
Non-adherent	2391	37.2	1586	46.6	3977	40.4					

AR = Aortic regurgitation; AS = Aortic stenosis; ASOT = Antistreptolysin-O titre; BPG = Benzathine penicillin G; ESR = erythrocyte sedimentation rate; MR = mitral regurgitation; MS = mitral stenosis; RHD = rheumatic heart disease; TR = tricuspid regurgitation

### Recurrent attacks of tonsillitis and tonsillectomy

**Fig 5:**
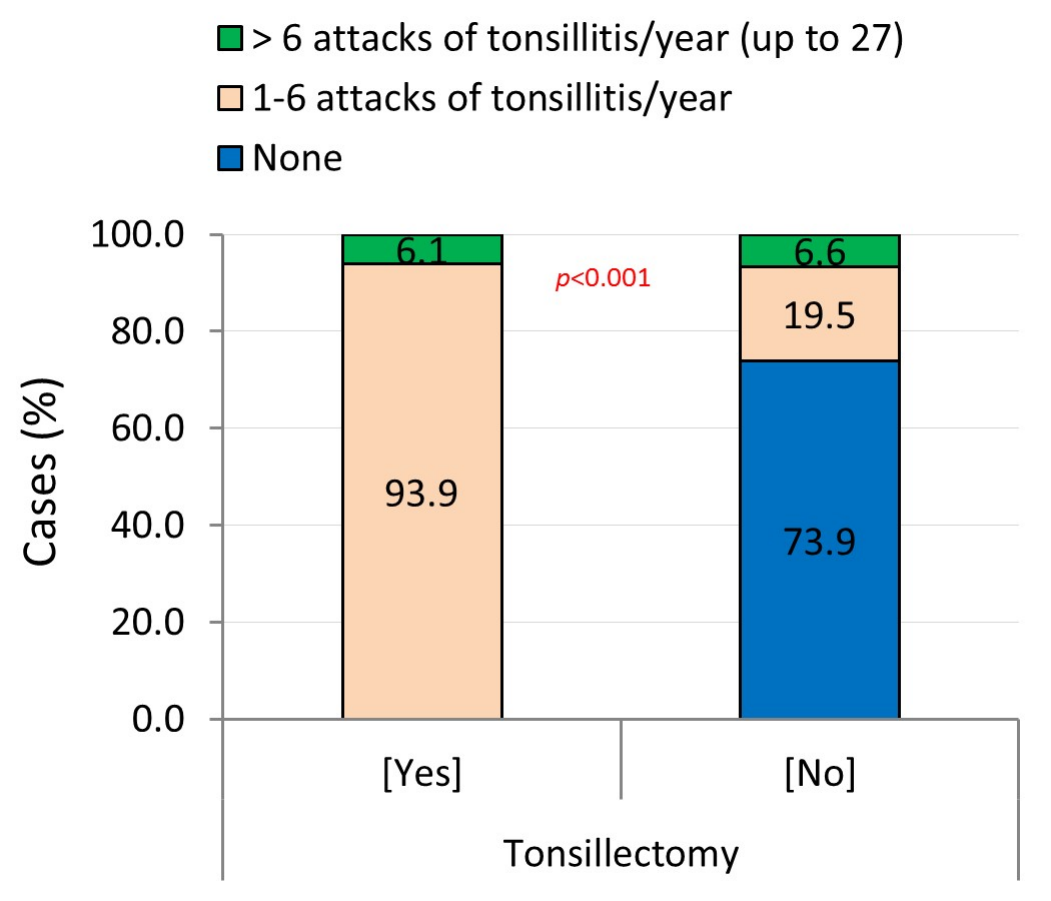
Tonsillectomy among cases with a history of recurrent attacks of tonsillitis attending the El-Mahalla RHD centre (2006–2018).

Rheumatic patients were more likely to experience recurrent attacks of tonsillitis (59.3%; ranged in number between 1–27 attacks per year) and eventually underwent tonsillectomy (65.2%) (Table 1). A considerable number of the latter eventually developed ARF complications [carditis/RHD (10.8%), Rh.A (19.2%), carditis/RHD and Sydenham’s chorea (0.1%), and combined carditis/RHD and Rh.A (2.2%)] (S1 Table). In most tonsillectomy patients (93.9%), the frequency of tonsillitis was less than 6 attacks per year (Fig 5).

### Use of BPG

Almost 58.0% of the study cohort was receiving BPG injections as ARF secondary prophylaxis, mainly on biweekly basis (83.8%), although only 53.4% of them adhered to the regimen. The use of a 3-week or a 4-week BPG regimens and the lack of compliance with its use were remarkable features among rheumatic patients compared to their normal peers (*p*< 0.05). Almost one-third of those who reported receiving BPG injections as ARF secondary prophylaxis eventually developed ARF complications (12.2% carditis/RHD, 20.0% Rh.A, 0.05% carditis/RHD and Sydenham’s chorea, and 2.3% carditis/RHD and Rh.A) (S2 Table). The use of BPG was more common among patients experiencing recurrent attacks of tonsillitis, although the non-adherence rate and rate of administration of BPG injections every 3^rd^ or 4^th^ week were significantly higher in these patients compared to those with no frequent tonsillitis (S3 Table).

### Echocardiography of rheumatic patients

In total, 12.6% of the screened subjects had clinical evidence of cardiac valvular damage due to ARF. Valvular involvement was predominately mitral (89.8%), mild (76.5%) and regurgitant (91.3%). The majority of RHD patients had mitral regurgitation (81.4%) and less frequently had mitral stenosis (11.3%), tricuspid regurgitation (10.9%), aortic stenosis (10.3%), or aortic regurgitation (1.9%). Triple and double valvular involvements did not exceed 1.5% (Table 2). The degree of valvular affection and the associated factors are detailed in S4 Table.

### Misdiagnosis of ARF and its sequelae

We ruled out ARF/RHD in a significant number (37.7%) of a group of the participants who were previously misdiagnosed as rheumatic patients and were un-necessarily on BPG secondary prophylaxis regimens as recommended by their health care providers. Misdiagnosed cases tended to present with breathlessness/dyspnoea on effort (which significantly correlated with smoking history) (60.7%), tonsillitis (52.5%), arthralgia (7.9%) or other non-specific symptoms (0.9%) (*p* <0.05) (Table 3).

**Table 3:** Factors associated with misdiagnosis of rheumatic fever and its sequelae.

	Misdiagnosis				Univariate analysis	Multivariate analysis			
	Yes (n = 6411)		No (n = 10603)			Exp(B)	95% C.I. for EXP(B)		*p*
	n	%	n	%	*p*		LL	UL	
**Age (Years)**						** **			
<5	132	2.1	617	5.8	**<0.001**				**<0.001**
5 –<15	3752	58.5	7243	68.3		0.72	0.42	1.21	0.212
15 –<30	2013	**31.4**	2090	19.7		**0.44**	**0.24**	**0.78**	**0.005**
30–50	505	**7.9**	584	5.5		**0.38**	**0.20**	**0.72**	**0.003**
50+	22	0.2	56	1.2		**0.03**	**0.01**	**0.07**	**<0.001**
**(Mean ± SD)**	12.6 ± 8.6		14.9 ± 8.4		**<0.001**				
**Smoking**									
Yes	166	2.6	229	2.2	**0.032**	**0.57**	**0.40**	**0.81**	**0.002**
passive	1586	24.7	2349	22.2	**<0.001**	0.95	0.82	1.10	0.485
**Sex**									
Male	2136	33.3	4123	38.9	**<0.001**				
Female	4275	66.7	6480	61.1					** **
**Residence**					** **				
Rural	4157	64.8	6259	59.0	**<0.001**	**1.20**	**1.10**	**1.40**	**0.001**
Urban	2254	35.2	4344	41.0					
**Education**					** **				
Illiterate	162	2.5	242	2.3	**<0.001**	0.98	0.64	1.49	0.907
Read and Write	198	3.1	351	3.3		0.83	0.57	1.21	0.334
Kindergarten	480	7.5	1727	16.3		**0.52**	**0.37**	**0.71**	**<0.001**
Primary	2485	38.8	4835	45.6		**0.68**	**0.48**	**0.95**	**0.026**
Preparatory	1180	18.4	1562	14.7		**0.72**	**0.53**	**0.99**	**0.044**
Secondary	1451	22.6	1431	13.5		0.87	0.69	1.09	0.226
University	613	5.0	297	6.4					**<0.001**
**Father's occupation**									
Working	128	2.1	234	2.3					
Not working	5664	94.2	9492	94.1	**0.002**				
Retired	220	3.7	357	3.5					
**Mother's occupation**					** **				
Working	5447	86.0	8969	85.6					
Not Working	888	14.0	1511	14.4	0.763				** **
**Parents**									
Alive	5936	92.6	9960	93.9	**<0.001**				
Died	475	7.4	643	6.1					
**Marital status**					** **				
Single	1113	52.6	1218	52.4	**<0.001**	1.04	0.80	1.35	0.774
Married	977	46.2	1081	46.5		**1.40**	**1.13**	**1.74**	**0.002**
Widowed	10	0.5	8	0.3		2.93	0.80	10.71	0.104
Divorced	16	0.8	16	0.7		0.98	0.33	2.90	0.971
**Income**									
Not enough	6125	49.6	2411	51.6	**0.022**				
Enough	6216	50.4	2262	48.4		1.60	1.40	1.80	**<0.001**
**Family Size**					** **				
1–3	456	7.1	570	5.4					0.001
4–8	5880	91.7	9912	93.5	**<0.001**	**0.71**	**0.56**	**0.90**	**0.004**
>8 (9–15)	75	1.2	121	1.1		**0.44**	**0.28**	**0.72**	**0.001**
**(Mean ± SD)**	5.03 ± 1.2		5.02 ± 1.18		0.511	0.92	0.88	0.97	0.001
**Crowding Index**					** **	** **	** **	** **	
1–2	6175	96.3	10176	96.0	0.474				
3–5	206	3.2	378	3.6					** **
>5 (6–11)	30	0.5	49	0.5					** **
**(Mean ± SD)**	1.58 ± 0.66		1.6 ± 0.7		0.062	**1.20**	**1.09**	**1.32**	**<0.001**
**Ventilation**					** **				
Good	4588	71.6	7238	68.3		1.885	1.344	2.643	**<0.001**
Average	1654	25.8	3036	28.6	**<0.001**	1.19	0.86	1.65	0.301
Bad	169	2.6	329	3.1		** **	** **	** **	**<0.001**
**Medical service**					** **				
None	100	1.6	3934	37.1	**<0.001**	**0.05**	**0.03**	**0.07**	**<0.001**
Private health care services	5736	89.5	3389	32.0		**1.42**	**1.03**	**1.95**	**0.031**
Health Insurance	392	6.1	2412	22.7		1.20	.84	1.72	0.315
PHC units	55	0.9	564	5.3		1.17	.65	2.10	0.595
Public Hospitals	128	2	304	2.9					**<0.001**
**Referral method**									
Health care Physician	6369	99.3	8489	80.1	**<0.001**	**0.09**	**0.02**	**0.50**	**0.006**
Family	12	0.2	644	6.1		0.92	0.06	14.2	0.954
Advertisement	13	0.2	974	9.2		0.17	0.02	1.43	0.093
Friend	1	0.0	3	0.0		0.16	0.01	4.51	0.284
Neighbours	4	0.1	427	4.0		**0.02**	**0.002**	**0.19**	**0.001**
Others	12	0.2	66	0.6					**0.004**
Symptoms					* *				
Tonsillitis	3367	52.5	5510	52.0	0.484				** **
Pharyngitis	479	7.5	943	8.9	**0.001**	**0.75**	**0.62**	**0.89**	**0.001**
Breathlessness/Dyspnea on effort	3890	60.7	5431	51.2	**<0.001**	**1.21**	**1.07**	**1.37**	**0.004**
Arthralgia	454	7.1	520	4.9	**<0.001**	**5.51**	**3.50**	**8.68**	**<0.001**
Arthritis	427	6.7	2131	20.1	**<0.001**	**0.08**	**0.07**	**0.10**	**<0.001**
Others	59	0.9	84	0.8	0.376	**8.96**	**3.56**	**22.5**	**<0.001**
Recurrent attacks of tonsillitis									
No						2.40	1.94	2.96	**<0.001**
Yes	3261	50.9	5311	50.1	0.327				
≤ 6 attacks per year	2928	89.8	4547	85.6	**<0.001**	1.88	1.54	2.29	**<0.001**
> 6 attacks per year (up to 27)	333	10.2	764	14.4		**0.42**	**0.34**	**0.52**	**<0.001**
**Major criteria of Rheumatic fever**									
Carditis	0	0.0	2138	20.2	**<0.001**				
Arthritis	0	0.0	2835	26.7					
Sydenham’s Chorea	0	0.0	8	0.1					
**Minor criteria of Rheumatic fever**									
ASOT (Mean ± SD)	415.4 ± 165.8		434.8 ± 173.6		0.220				
ESR 1st hr (Mean ± SD)	21.3 ± 13.5		22.8 ± 15.7		0.320				
**Tonsillectomy**									
Yes	2256	35.2	3330	31.4	**<0.001**				
**Hospitalization**									
Cardiac	29	0.5	125	1.2	**<0.001**	**0.28**	**0.17**	**0.47**	**<0.001**
Others	414	6.5	829	7.8		0.90	0.74	1.11	0.337
**History of taking BPG**					ND				
Yes	6411	100.0	3425	32.3					
**BPG regimen**									
2 weeks	5595	87.3	2869	83.8	**<0.001**				
3 weeks	158	2.5	96	2.8					
4 weeks	658	10.3	460	13.4					
**BPG Frequency**									
Adherent	4023	**62.8**	1836	53.6	**<0.001 **	**1.45**	**1.28**	**1.63**	**<0.001**
Non-adherent	2388	37.2	1589	46.4					

ASOT = Antistreptolysin-O titre; BPG = Benzathine penicillin G; ESR = erythrocyte sedimentation rate; PHC = primary health care

### Rheumatic fever among family members of RHD patients

Screening of family members of RHD patients who had valve replacement cardiac surgery, resulted in the detection of 20.7% rheumatic cases [56.0% with RHD and 52.2% with Rh.A]. These represented 3.4% of the total cases diagnosed with ARF and its complications (S5 Table).

### Multivariate analysis: ARF/RHD risk model

In multivariate stepwise conditional logistic regression analyses, there was a trend towards acquiring ARF or developing RHD in association with age group 15 –<30 years, female sex, urban residence, passive smoking, being in kindergarten, primary or preparatory school, having an unemployed mother, having a low income, having a large family size, a crowding index from 3 to 5 or >5, living in average and poorly ventilated households; having no access to health care services, and being referred by a health care physician (Table 1). Rheumatic patients notably presented with repeated attacks of tonsillitis [OR  =  1.31, 95% CI  =  1.2–1.42] and arthritis [OR  =  11.0, 95% CI  =  9.7–12.4], and experienced more frequent hospitalization due to cardiac causes. Adherence to BPG prophylaxis apparently reduced ARF/RHD rates [OR  =  0.75, 95% CI  =  0.67–0.84] (Table 2).

On the other hand, misdiagnosed cases showed different sociodemographic features. Particularly, rural residence, being married, having enough income, having a small family size, having a high crowding index, living in a well-ventilated house, and utilizing private health care services. They initially presented with dyspnoea [OR  =  1.21, 95% CI  =  1.07–1.37], arthralgia [OR  =  5.5, 95% CI  =  3.5–8.68], or non-specific symptoms [OR  =  8.9, 95% CI  =  3.6–22.5], but never experienced recurrent attacks of tonsillitis [OR  =  2.4, 95% CI  =  1.9–2.96] and tended to have fewer than 6 attacks per year [OR  =  1.9, 95% CI  =  1.5–2.3]. Moreover, they never been hospitalized due to cardiac causes [OR  =  0.28, 95% CI  =  0.17–0.47], and were adherent to PBG use [OR  =  1.45, 95% CI  =  1.28–1.63] (Table 3).

Likewise, family members of RHD patients had a higher mean family size [OR = 1.3, 95% CI =  1.07–1.57], a higher crowding index [OR = 8.6, 95% CI = 1.01–72.9], no access to health care services [OR = 3.3, 95% CI = 1.2–9.5], and presented with pharyngitis [OR = 2.04, 95% CI =  1.05–4.0] or arthritis [OR = 11.4, 95% CI =  6.6–19.8] (S5 Table).

## Discussion

The present data paint the unabated picture of rheumatic fever in Egypt and the heavy economic burden placed by RHD on the healthcare system. However, with the expansion of the national rheumatic heart disease prevention and control program screening capacity, the proportion of the identified rheumatic patients consistently declined over time. Furthermore, more ARF/RHD misdiagnoses were spotted and correctly diagnosed. This reflects the effectiveness of the program in achieving its objectives, particularly after gaining more national attention and governance from the Egyptian MOHP starting from the year 2014 onwards.

### Characteristics of the screened cohort

The incidence of ARF is highest in children aged 5–15 years [3]. This was consistent with findings from our study cohort, although we reported cases of ARF in patients as young as 2–4 years old. Children in the 5- to 15-year-old age group still have the highest risk for ARF in Egypt because at these ages, children are in schools that are badly ventilated and overcrowded; conditions that favour the spread of GABHS infections [31]. This particular age-group vulnerability was suggested to be attributed to delayed antibody response and maturation processes in the immune system [32]. We identified ARF in people older than 30 years. Although we could not correctly delineate whether these were initial or recurrent episodes, literature would suggest these cases are almost entirely recurrences [1,4]. RHD is caused by accumulated heart valve damage from recurrent ARF episodes. However, the peak of its prevalence was observed among children in the age group 5–15 years, although RHD usually occurs in adulthood between the ages of 25 years and 45 years [33]. This suggests that children frequently do not have their initial bout of ARF come to medical attention. In this context, children may be misdiagnosed with initial ARF when they are, in fact, experiencing a recurrence of ARF. The intensification of awareness-raising activities in recent years may help to reduce the extent of this problem in the future.

In most populations, ARF is equally common in males and females, although an increased risk of RHD in females has been found in almost all populations [3,4,31,33,34], particularly in adolescents and adults rather than in children [26,33]. Conversely, male sex predominance of valvular disease in ARF was previously reported in Nepali children [35]. However, in the present cohort, all rheumatic presentations were more common in females, with a relative risk of 1.2 compared with males. For instance, the reason for this sex difference is not clear but could be attributed to intrinsic factors such as greater autoimmune susceptibility in females (influence of oestrogen on B cell response, T lymphocyte proliferation, profibrotic Th2 response and level of inflammatory cytokines) [36,37], or extrinsic factors such as greater exposure to repeated GABHS infections in women than in men. It is likely that women are housebound, spending longer times engaged in indoor activities and exposes more to overcrowding under poor housing conditions. Moreover, in low social classes, health care seeking for women is constrained by sociocultural norms, and thus they get inequitable access to health care [38]. The delay in the diagnosis of mild to moderate episodes of ARF among women may ultimately lead to a large population of female patients with RHD [4]. Likewise, RHD is increasingly recognized during antenatal care and was reported as a leading cause of indirect obstetric death [39].

Tackling the socioeconomic and environmental determinants of ARF is crucial in elucidating the disease epidemiology [3,11]. Most social determinants of health are overlapping and their association with poverty and economic disadvantages are well documented [26]. Household overcrowding is the most important factor underlying the high ARF incidence [40,41]. With a higher family size in the evaluated participants, there was a notable increase in the rate of ARF and its sequelae, which may reflect the effect of household crowding and deprivation [4,40,42]. This was consistent in previous reports from around the world [3,4,9,10,11,31,33,34,41,42]. Likewise, a higher crowding index and poor ventilation obviously affected the occurrence of ARF and its complications in this cohort. In fact, ventilation is a key pillar of infection control in buildings thanks to the germicidal effect of direct sunlight [43,44,45], and poor living conditions and deteriorated indoor environment could potentiate the transmission of bacterial pathogens such as GABHS. This was consistently documented in many studies [3,4,31,33,34,41,46] except for a single report from India that found no clear difference in RHD occurrence between children living in homes with a higher crowding index and those living in less crowded households [47].

ARF and RHD were found to be more common in patients from rural locations [3,4], although in some studies, the risk was highest in impoverished urban regions, with overcrowding and poor housing conditions [48,49]. Many Egyptian villages, including El-Mahalla, have experienced rapid urbanization, which has brought a population shift from rural to urban locations, leading to congested socioeconomically depressed localities. This clarifies why urban residence in the present cohort correlated with ARF and RHD. Furthermore, one-third of rheumatic patients had no access to health care services. In fact, the distance to a health centre can delay access to prophylactic medication and is an obstacle to adherence to regular BPG injections, a critical component of ARF secondary prevention [41].

Low income was a noteworthy feature of rheumatic patients in the present study. ARF and RHD are diseases of poverty and can emerge in settings of social disruption [50,51,52,53,54]. Children of unemployed mothers were found to be at greater risk of both ARF and RHD. In studies conducted in Fiji [54] and Uganda [41], maternal unemployment was a robust independent predictor of RHD. For instance, unemployment was a confounder of low literacy among mothers of rheumatic children. This link can be viewed as the effect of quality of care by educated mothers, awareness of simple ways to fight the disease, and appropriate use of health services [4]. Moreover, unemployment has economic implications. The program in El-Mahala implements daily health education sessions targeting mothers and caregivers of children attending the centre for screening or follow-up of their condition. The key messages are the association between throat infections and ARF, and the importance of completing the antibiotic course to prevent the disease and its consequent complications.

In the present study, exposure to second-hand smoking was more encountered by the rheumatic patients than normal individuals. Plausibly, environmental tobacco smoke is an established risk factor for respiratory infections and may initiate ARF in susceptible individuals [55] by altering oropharyngeal flora and increasing bacterial pathogens, including GABHS [56]. In New Zealand, 71% of ARF patients lived in homes with at least one smoker [57].

### Clinical presentation and cardiac sequelae

The World Heart Federation Rheumatic Heart Disease Program reported that arthritis is the most common symptom in up to 75% of first episodes of ARF [8]. This agrees with the presentations in our cohort. Mitral stenosis is common at the time of initial presentation in the developing world [58] because many patients seek medical attention too late [59]. However, the RHD control program in El-Mahalla succeeded in the early detection of cases of RHD before severe valvular involvement has occurred. Consistent with other local [31,34] and worldwide [26,35,60,61] studies, the majority of the RHD cases had mild mitral regurgitation. Trivial valvular effects were recorded for 241 (11.3%) patients who were counselled regarding the appropriate duration of secondary prophylaxis and advised to attend regular follow-up visits to prevent progressive damage to their valves.

Sydenham’s chorea occurs in up to 30% of those who have ARF and is strongly associated with carditis [3]. However, in our study cohort, it was an ARF presentation in less than 0.05% of cases, but was combined with carditis in 75% of the cases. This may reflect a noteworthy change in the clinical pattern of ARF.

### Recurrent attacks of tonsillitis and tonsillectomy

Recurrent attacks of tonsillitis/pharyngitis were common among rheumatic patients compared to normal individuals. Similarly, Bassili et al. found that a history of tonsillitis was reported by one-third of the studied children [62]. This is plausible since tonsillitis is a source of GABHS, which is implicated in the pathogenesis of ARF [63,64]. Although most of the children experiencing repeated attacks of tonsillitis were advised to undergo tonsillectomy before the study, an appreciable number eventually developed ARF in its different presentations. In the Egyptian community, families make the decision to undergo tonsillectomy as a measure to prevent frequent respiratory infections or repeated attacks of tonsillitis and the attendant complications, although ARF and its sequelae may have been already established. Importantly and from the authors’ experience, people who have their children tonsillectomized do not seek treatment for subsequent attacks of pharyngitis because they believe that tonsillectomy will interrupt the infection and protect against ARF and RHD. This reflects a low level of community awareness of the disease and inadequate expertise from the part of health care providers. There is strong evidence that tonsillectomy does not alter the susceptibility to infection with GABHS or its sequelae [65,66,67,68,69]. Once that infection has been established, the presence or absence of the tonsils does not alter the clinical course of the disease. Moreover, it appears that streptococcal infections are less readily recognized in tonsillectomized patients and, therefore, are more likely to escape treatment appropriate for the prevention of ARF [67,69].

### Use of BPG

We were not able to obtain accurate data about the primary prophylaxis of ARF as initially received by the participants for treating GABHS related diseases. As a matter of fact, the local practice of antibiotic treatment for children with pharyngitis and fever relies on self-medication with oral antibiotics dispensed from community pharmacies without prior consultations, appropriate prescriptions or indications [70,71,72,73]. Moreover, some practitioners rely on a constellation of clinical features for presumptive treatment of streptococcal pharyngitis since bacterial culture and rapid diagnostic tests are not feasible [74].

As secondary prophylaxis for ARF, the majority of the studied children reported receiving BPG injections mainly as a biweekly regimen, although half of them were not compliant with the regimen largely due to pain associated with the injection or a shortage of medicine supply in health care units. A 28-day BPG prophylaxis for at least 10 years was found to be safe and effective for patients with no or mild cardiac disease [5,6]. Nevertheless, in agreement with the findings of Lue and co-workers [75], patients receiving BPG every third/fourth week or the lack of compliance were strongly associated with the development of ARF or its recurrence. A Cochrane meta-analysis confirmed that injections every two or three weeks were more effective than 4-weekly regimens, although the evidence was based on poor quality trials [76]. Studies on the serum penicillin levels in Egyptian children have shown a drop in the serum penicillin concentration to below the therapeutic level during the third week following the injection [62,77]. Moreover, the difference in the pharmacokinetics of locally manufactured BPG compared to brand penicillin was found to contribute to the high recurrence rate of ARF reported in Egypt [77,78]. Consequently, the national guidelines endorse the use of biweekly regimens for Egyptian patients until work on strengthening the BPG quality is successfully accomplished.

In the present report, the use of BPG as secondary prophylaxis for ARF did not reduce the risk of developing ARF related sequelae. Indeed, the treatment of existing GABHS infections may decrease the overall damage from that episode, but it will not eliminate or stop the progression of damage from the initial infection [79]. Furthermore, one-third of ARF episodes results from non-symptomatic GABHS infections that go unnoticed and untreated [29]. In community settings, the evidence to support injectable or oral penicillin treatment of GABHS pharyngitis to prevent the first presentation of ARF or decrease its frequency is still controversial [3]. Prompt treatment of GABHS pharyngitis was postulated to remove the pathogen before an effective and prolonged immune response is elicited, leaving the host more susceptible to recurrent infections [80,81]. However, this theory has been challenged in other studies [82,83].

The efficacy of BPG as a secondary prophylaxis for ARF is limited by low patient adherence. A systematic, generalizable tool was developed by Balbaa and co-workers to outline and ultimately address these barriers. Adherence deterrents were the clinic wait time and the lack of knowledge of the nature of the disease or the consequences of missing prophylaxis doses [84]. The delivery of secondary antibiotic prophylaxis should be performed by a dedicated health care team. Physician attention and informed discussions regarding treatment efficacy and life events empower people to take responsibility for their own wellbeing and contribute to better patient adherence [79]. Maintaining adherence to BPG secondary prophylaxis might be prevented by physicians seeking to limit penicillin use in order to alleviate the development of antibiotic resistance. This highlights the importance of primary prevention through socioeconomic development, access to medical care, and reducing the prevalence of streptococcus in the environment. Another potential path to the primary prevention of ARF and to obviate the inherent BPG disadvantages is the development of a GABHS vaccine which is currently underway [85,86].

### Misdiagnosis of ARF and its sequelae

The diagnosis of ARF remains challenging due to the changing pattern of the disease with a variety of clinical manifestations. In the present study, more than one-third of the evaluated participants that were previously diagnosed with ARF/RHD were found to be misdiagnosed and were regularly receiving unnecessary BPG. This undoubtedly increases the potential for adverse antibiotic reactions and creates excessive parental anxiety. The majority of the screened subjects, including the misdiagnosed patients, were referred to the El-Mahala RHD centre by health care practitioners in the private sector, who lack training and access to echocardiography.

As a matter of fact, ARF is a neglected disease, and physicians are only vaguely aware of the disorder. In this regard, capacity building, empowerment, and far greater awareness-raising endeavours amongst Egyptian health professionals are needed. Meanwhile, there is no biological marker for ARF, and its diagnosis is based on a constellation of clinical (Jones criteria) [15] and laboratory evidence of previous streptococcal infections. However, the established criteria needed to make a diagnosis might not arise concurrently, and the initial illness may be mild and short-lived. This may result in underdiagnosis or overdiagnosis [87]. The diagnosis will be missed if appropriate investigations are not carried out during the acute illness. On the other hand, too much reliance on elevated ASOT as a biomarker for GABHS throat infections preceding ARF or elevated acute phase reactants can result in unnecessary treatment. Likewise, the isolation of GABHS from a patient with tonsillitis/pharyngitis does not demonstrate a causal relationship. Not all individuals experiencing an untreated GABHS infection develop ARF. Moreover, not all strains of GABHS trigger an acute attack even in a highly susceptible host [88].

Misdiagnosed patients were receiving un-necessary BPG on a regular basis, and during the interview, they named it “Hoknet Al-Tarseeb” in colloquial Arabic [where; Hoknet = injection, Al = the, and Tarseeb = sedimentation or precipitation], referring by that to an injection that is given when the ESR is high. This means that the diagnosis was incorrectly made by untrained practitioners on the basis of the elevated ESR when the patient initially presented with symptoms suggestive of ARF, without confirming or referring the suspected case to a specialized RHD clinic. An elevated ESR should alert a physician that ARF is likely when articular symptoms are observed, even in the absence of clinical carditis and before the echocardiologic findings can be reviewed [89]. The possibility of misclassification in established RHD has increased in recent years, due to poor clinicians’ auscultatory skills [6]. Common sources of error in the diagnosis of ARF in children can include interpreting innocent murmur as the murmur associated with RHD; arthralgia as arthritis; abnormal movements as chorea; low-grade, persistent fever as ARF; clinical evidence of sore throat as GABHS infection (a variety of bacterial and viral agents can be implicated); and elevated ASOT as evidence of ARF [90]. Furthermore, in many high-risk tropical areas, acute onset arthritis and fever are commonly caused by arboviral infections that should be considered and evaluated by testing whenever possible [91].

Obviously, the results revealed that misdiagnosed cases had different sociodemographic and clinical features compared to rheumatic cases. They were more likely to present with breathlessness/dyspnoea or arthralgia rather than arthritis. Probably, breathlessness was a result of anaemia or an adverse effect of smoking in a number of these patients. Physicians must adhere to the revised Jones criteria [15] for the diagnosis of ARF because atypical joint involvement and silent carditis can occur particularly in endemic areas, which can lead to misdiagnosis or delayed diagnosis and eventually ends in valvular damage [15,22,89,92]. Echocardiography is the gold standard in swiftly confirming or ruling out acute carditis or established valvular involvement in questionable cases [93].

To reduce misdiagnoses in the future, our study supports that the work-up for ARF should include an echocardiogram and that its finding should be scored as a major criterion for the diagnosis of RHD. Physician education and clear revised guidelines are necessary to ensure adequate management of individuals with ARF.

### Risk ARF and RHD in family members

RHD is known to cluster in families although it cannot be transmitted directly from parents to their children. The molecular basis of ARF and the subsequent RHD remains poorly understood [3,7]. As inferred from the present study, children of RHD patients are prone to acquire ARF and develop RHD compared to their peers in the community borne to parents not suffering from the disease. Similar findings were reported among school children in sub-Saharan Africa [94]. This suggests a familial tendency or genetic predisposition to the disease that can be aggravated by social factors and environmental conditions shared by inhabitants of the same household and favouring the occurrence of ARF. The genetic susceptibility to ARF and RHD was well documented in certain countries, including Australia, India, Egypt, and Turkey [3,4,95,96,97], but no work to show this has been done in African countries. Susceptibility to ARF was found to be genetically determined, supported by phenotypic concordance among dizygotic twins [95] and linked to polymorphisms in several genes coding for immune-related proteins [3,96,98,99,100]. Apparently, susceptibility to ARF and RHD are polygenic and potentially inked to HLA loci, B-cell alloantigens, and immune-modulatory genes. Specific HLA class II haplotype conferred strong protection against ARF [96,98,99,100], whereas others increased the risk of rheumatic disease in Egyptian [63,101], Latvian [98,99] and Taiwanese children [102]. Moreover, a B cell alloantigen (D8/17) was detected in rheumatic patients and their family members [3]. Whole-genome studies of susceptibility to ARF and RHD are anticipated in a number of high-incidence settings, which may provide further insights in the coming years [96,97].

Admittedly, genetics differ among different populations and are not strong enough to indicate a need for screening and primary prophylaxis. But having a family history of ARF or RHD critically changes the pre-test probability of a diagnosis of rheumatic fever in someone presenting with suggestive symptoms, as was recently introduced in the revised Jones criteria algorithm for high-risk individuals [15].

### Study limitations

Limitations in the current report entail the use of health program based consecutive sampling instead of community-based sampling which would provide more precise prevalence data. Thus, the present data appear as a surrogate to describe the magnitude of ARF/RHD in the referral communities. Besides, our investigation did not focus on finding new factors that might pose an increased risk, rather looked for the recognized risk factors and how these operate in the Egyptian population. Data about housing conditions were based on interviewing the participants and have not been ascertained through household visits due to time and resource constraints.

## Conclusions

Collectively, ARF continues to occur in Egypt and is often associated with cardiac affection. The disease is enduring in impoverished urban regions in Egypt that are linked with underprivileged socioeconomic determinants and poorly ventilated premises. Misdiagnosis of ARF and its complications is noticeably common. This, together with poor compliance to BPG prophylaxis and poor living conditions, may affect efforts being exerted to eradicate the disease.

Children of RHD patients seem to have a higher predilection for ARF/RHD. Accordingly, we emphasize that suspicion of ARF/RHD should be heightened when there is a family history. Screening of family members of RHD patients is warranted if they reside in high-risk environments.

The messages that came from our experience have potentially important implications for echocardiography-based case finding and early detection, delivery of effective prevention, and adequate planning of health services.

### Priority for action

Sustained outreach RHD screening campaigns and school-based clinics employing echocardiography should be established in marginalized communities for the early detection of the disease.

Formulating national guidelines, enforcing a well-planned national strategy, upgrading the national registry system by employing REDCap (Research Electronic Data Capture), capacity building, increased access to echocardiography, strengthening the quality of BPG for better patient compliance, fostering research for new streptococcal vaccine candidates, and adopting appropriate investigations should be emphasized.

## Supporting information

S1 Table Regimens of Benzathine Penicillin G in primary and secondary prophylaxis of rheumatic fever in Egypt. (DOCX)

S2 Table Final diagnosis in relation to recurrent attacks of tonsillitis, tonsillectomy and use of BPG for ARF secondary prophylaxis. (DOCX)

S3 Table Recurrent attacks of tonsillitis in relation to history of tonsillectomy and use of BPG for ARF secondary prophylaxis. (DOCX)

S4 Table Degree of valvular involvement and the associated factors among rheumatic patients. (DOCX)

S5 Table Factors associated with of rheumatic fever and its sequelae among family members of RHD patients who had valve replacement surgery. (DOCX)

S1 Fig Distribution of rheumatic fever cases clinically presenting with carditis/RHD or rheumatic arthritis per year in El-Mahalla RHD centre (2006–2018). (TIF)

S2 Fig Seasonal pattern of rheumatic fever and its sequelae in Egypt. (TIF)

S1 Video Folkloric song for children promoting health message for rheumatic fever and rheumatic heart disease prevention. (MP4)

## References

[1] Cunningham MW (2016) Post-streptococcal autoimmune sequelae: Rheumatic fever and beyond. 26866235

[2] Zuhlke LJ, Beaton A, Engel ME, Hugo-Hamman CT, Karthikeyan G, et al (2017) Group A streptococcus, acute rheumatic fever and rheumatic heart disease: Epidemiology and clinical considerations. Curr Treat Options Cardiovasc Med 19: 15 10.1007/s11936-017-0513-y 28285457 PMC5346434

[3] Carapetis JR, Beaton A, Cunningham MW, Guilherme L, Karthikeyan G, et al (2016) Acute rheumatic fever and rheumatic heart disease. Nat Rev Dis Primers 2: 15084 10.1038/nrdp.2015.84 27188830 PMC5810582

[4] Baker MG, Gurney J, Oliver J, Moreland NJ, Williamson DA, et al (2019) Risk factors for acute rheumatic fever: Literature review and protocol for a case-control study in New Zealand. Int J Environ Res Public Health 16. 10.3390/ijerph16224515 PMC6888501 31731673

[5] Spinetto H, Lennon D, Horsburgh M (2011) Rheumatic fever recurrence prevention: a nurse-led programme of 28-day penicillin in an area of high endemnicity. J Paediatr Child Health 47: 228–234. 10.1111/j.1440-1754.2010.01942.x 21470327

[6] Bowen A, Currie B, Katzenellenbogen J, Marangou J, S N, et al (2020) The 2020 Australian guideline for prevention, diagnosis and management of acute rheumatic fever and rheumatic heart disease In: Currie B, Ralph A, Noonan S, editors. 3rd ed. Casuarina NT: Menzies School of Health Research.

[7] Sika-Paotonu D, Beaton A, Raghu A, Steer A, Carapetis J (2016) Acute Rheumatic Fever and Rheumatic Heart Disease. In: Ferretti JJ, Stevens DL, Fischetti VA, editors. Streptococcus pyogenes: basic biology to clinical manifestations Oklahoma City (OK).

[8] Remenyi B, Carapetis J, Wyber R, Taubert K, Mayosi BM (2013) Position statement of the World Heart Federation on the prevention and control of rheumatic heart disease. Nat Rev Cardiol 10: 284–292. 10.1038/nrcardio.2013.34 23546444

[9] World Health Organization (2018) Rheumatic fever and rheumatic heart disease. Seventy-first world health assembly Provisional agenda item 12.8. p. 1–6. Geneva: WHO.

[10] Seckeler MD, Hoke TR (2011) The worldwide epidemiology of acute rheumatic fever and rheumatic heart disease. Clin Epidemiol 3: 67 10.2147/CLEP.S12977 21386976 PMC3046187

[11] Watkins DA, Johnson CO, Colquhoun SM, Karthikeyan G, Beaton A, et al (2017) Global, regional, and national burden of rheumatic Heart disease, 1990–2015. N Engl J Med 377: 713–722. 10.1056/NEJMoa1603693 28834488

[12] Egyptian Ministry of Health and Population (2018) National RHD prevention and control Program.

[13] Armstrong C (2010) AHA guidelines on prevention of rheumatic fever and diagnosis and treatment of acute Streptococcal pharyngitis. American Family Physician 81: 346.

[14] Remenyi B, Wilson N, Steer A, Ferreira B, Kado J, et al (2012) World Heart Federation criteria for echocardiographic diagnosis of rheumatic heart disease—an evidence-based guideline. Nat Rev Cardiol 9: 297–309. 10.1038/nrcardio.2012.7 22371105 PMC5523449

[15] Beaton A, Carapetis J (2015) The 2015 revision of the Jones criteria for the diagnosis of acute rheumatic fever: implications for practice in low-income and middle-income countries. Heart Asia 7: 7–11. 10.1136/heartasia-2015-010648 PMC4832790 27326214

[16] Wyber R (2013) A conceptual framework for comprehensive rheumatic heart disease control programs. Glob Heart 8: 241–246. 10.1016/j.gheart.2013.07.003 25690502

[17] Central Agency for Public Mobilization and Statistics (CAPMAS) (2017) Population Clock. Cairo: CAPMAS.

[18] El-Gilany A, El-Wehady A, El-Wasify M (2012) Updating and validation of the socioeconomic status scale for health research in Egypt. East Mediterr Health J 18: 962–968. 10.26719/2012.18.9.962 23057390

[19] Melki IS, Beydoun HA, Khogali M, Tamim H, Yunis KA (2004) Household crowding index: a correlate of socioeconomic status and inter-pregnancy spacing in an urban setting. J Epidemiol Commun Health 58: 476–480. 10.1136/jech.2003.012690 PMC1732777 15143115

[20] WHO (1999) WHO model prescribing information: drugs used in the treatment of streptococcal pharyngitis and prevention of rheumatic fever. Geneva: World Health Organization.

[21] Musoke C, Mondo CK, Okello E, Zhang W, Kakande B, et al (2013) Benzathine penicillin adherence for secondary prophylaxis among patients affected with rheumatic heart disease attending Mulago Hospital. Cardiovasc J Afr 24: 124–129. 10.5830/CVJA-2013-022 24217043 PMC3721822

[22] Banna HH, Swelam RA (2013) Clinical presentations of atypical arthritis in Egyptian children with acute rheumatic fever. J Amr Sci 9: 253–261.

[23] Kotby AA, Habeeb NM, Ezz El Elarab S (2012) Antistreptolysin O titer in health and disease: levels and significance. Pediatr Rep 4: e8 10.4081/pr.2012.e8 22690314 PMC3357621

[24] El-magd EAA, Meguid MA, El Tahan AER (2016) The value of high antistreptolysin O titre as an indicator of tonsillectomy in Upper Egypt. Int J Otolaryngol Head Neck Surg 5: 1.

[25] Sethi S, Kaushik K, Mohandas K, Sengupta C, Singh S, et al (2003) Anti-streptolysin O titers in normal healthy children of 5–15 years. Indian Pediatr 40: 1068–1071. 14660838

[26] Rothenbuhler M, O'Sullivan CJ, Stortecky S, Stefanini GG, Spitzer E, et al (2014) Active surveillance for rheumatic heart disease in endemic regions: a systematic review and meta-analysis of prevalence among children and adolescents. Lancet Glob Health 2: e717–726. 10.1016/S2214-109X(14)70310-9 25433627

[27] Salem DN, O'Gara PT, Madias C, Pauker SG (2008) Valvular and structural heart disease: American College of Chest Physicians Evidence-Based Clinical Practice Guidelines (8th Edition). Chest 133: 593S–629S. 10.1378/chest.08-0724 18574274

[28] Cannon J, Roberts K, Milne C, Carapetis JR (2017) Rheumatic Heart Disease Severity, Progression and Outcomes: A Multi‐State Model. Journal of the American Heart Association 6: e003498 10.1161/JAHA.116.003498 28255075 PMC5523987

[29] Gerber MA, Baltimore RS, Eaton CB, Gewitz M, Rowley AH, et al (2009) Prevention of rheumatic fever and diagnosis and treatment of acute Streptococcal pharyngitis: a scientific statement from the American Heart Association Rheumatic Fever, Endocarditis, and Kawasaki Disease Committee of the Council on Cardiovascular Disease in the Young, the Interdisciplinary Council on Functional Genomics and Translational Biology, and the Interdisciplinary Council on Quality of Care and Outcomes Research: endorsed by the American Academy of Pediatrics. Circulation 119: 1541–1551. 10.1161/CIRCULATIONAHA.109.191959 19246689

[30] World Health Organization (1999) WHO model prescribing information: drugs used in the treatment of streptococcal pharyngitis and prevention of rheumatic fever. Geneva: WHO.

[31] Elamrousy DM, Al-Asy H, Mawlana W (2014) Acute rheumatic fever In Egyptian children: A 30-year experience in a tertiary hospital. J Pediatr Sci 6.

[32] Coburn AF (1936) Observations on the mechanism of rheumatic fever. Lancet 228: 1025–1030.

[33] Lawrence JG, Carapetis JR, Griffiths K, Edwards K, Condon JR (2013) Acute rheumatic fever and rheumatic heart disease: incidence and progression in the Northern Territory of Australia, 1997 to 2010. Circulation 128: 492–501. 10.1161/CIRCULATIONAHA.113.001477 23794730

[34] El-Aroussy W, El-Hagracy N, Fawzy H, Zaher S, Taha N, et al (2013) Prevalence of rheumatic valvular heart disease among Egyptian school children: an echocardiographic screening. Med J Cairo Univ 81.

[35] Rayamajhi A, Sharma D, Shakya U (2007) Clinical, laboratory and echocardiographic profile of acute rheumatic fever in Nepali children. Ann Trop Paediatr 27: 169–177. 10.1179/146532807X220271 17716444

[36] Fairweather D, Frisancho-Kiss S, Rose NR (2008) Sex differences in autoimmune disease from a pathological perspective. Amr J Pathol 173: 600–609. 10.2353/ajpath.2008.071008 PMC2527069 18688037

[37] Ngo ST, Steyn FJ, McCombe PA (2014) Gender difference in autoimmune diseases. Front Neuroendocrinol 35: 347–369. 10.1016/j.yfrne.2014.04.004 24793874

[38] Rizvi SF, Khan MA, Kundi A, Marsh DR, Samad A, et al (2004) Status of rheumatic heart disease in rural Pakistan. Heart 90: 394–399. 10.1136/hrt.2003.025981 15020513 PMC1768176

[39] Diao M, Kane A, Ndiaye MB, Mbaye A, Bodian M, et al (2011) Pregnancy in women with heart disease in sub-Saharan Africa. Arch Cardiovasc Dis 104: 370–374. 10.1016/j.acvd.2011.04.001 21798468

[40] Jaine R, Baker M, Venugopal K (2011) Acute rheumatic fever associated with household crowding in a developed country. Pediatr Infect Dis J 30: 315–319. 10.1097/INF.0b013e3181fbd85b 20948456

[41] Okello E, Kakande B, Sebatta E, Kayima J, Kuteesa M, et al (2012) Socioeconomic and environmental risk factors among rheumatic heart disease patients in Uganda. PLoS One 7: e43917 10.1371/journal.pone.0043917 22952810 PMC3428272

[42] Sharma N, Toor D (2019) Impact of socio-economic factors on increased risk and progression of rheumatic heart disease in developing nations. Curr Infect Dis Rep 21: 21 10.1007/s11908-019-0677-6 31044281

[43] Hobday RA, Dancer S (2013) Roles of sunlight and natural ventilation for controlling infection: Historical and current perspectives. J Hosp Infect 84. 10.1016/j.jhin.2013.04.011 PMC7132476 23790506

[44] Hobday R (2013) The influence of sunlight and ventilation on indoor health: infection control for the post-antibiotic era.

[45] Clarke A (2019) Harper's practical genetic counselling: CRC Press.

[46] Vlajinac H, Adanja B, Marinkovic J, Jarebinski M (1991) Influence of socio-economic and other factors on rheumatic fever occurrence. Eur J Epidemiol 7: 702–704. 10.1007/BF00218687 1783067

[47] Baro L, Sharma N, Toor D, Chaliha MS, Kusre G, et al (2018) A hospital-based study of socioeconomic status and clinical spectrum of rheumatic heart disease patients of Assam, North-East India. Eur J Prev Cardiol 25: 1303–1306. 10.1177/2047487318787333 29984594

[48] Steer AC, Carapetis JR, Nolan TM, Shann F (2002) Systematic review of rheumatic heart disease prevalence in children in developing countries: the role of environmental factors. J Paediatr Child Health 38: 229–234. 10.1046/j.1440-1754.2002.00772.x 12047688

[49] Riaz BK, Selim S, Karim MN, Chowdhury KN, Chowdhury SH, et al (2013) Risk factors of rheumatic heart disease in Bangladesh: a case-control study. J Health Popul Nutr 31: 70–77. 10.3329/jhpn.v31i1.14751 23617207 PMC3702361

[50] Adanja B, Vlajinac H, Jarebinski M (1988) Socioeconomic factors in the etiology of rheumatic fever. J Hyg Epidemiol Microbiol Immunol 32: 329–335. 3198913

[51] Grover A, Dhawan A, Iyengar SD, Anand IS, Wahi PL, et al (1993) Epidemiology of rheumatic fever and rheumatic heart disease in a rural community in northern India. Bull World Health Organ 71: 59–66. 8440039 PMC2393425

[52] Zaman MM, Yoshiike N, Chowdhury AH, Jalil MQ, Mahmud RS, et al (1997) Socio-economic deprivation associated with acute rheumatic fever. A hospital-based case-control study in Bangladesh. Paediatr Perinat Epidemiol 11: 322–332. 10.1111/j.1365-3016.1997.tb00011.x 9246693

[53] Brown A, McDonald MI, Calma T (2007) Rheumatic fever and social justice. Med J Aust 186: 557–558. 17547542 10.5694/j.1326-5377.2007.tb01052.x

[54] Dobson J, Steer AC, Colquhoun S, Kado J (2012) Environmental factors and rheumatic heart disease in Fiji. Pediatr Cardiol 33: 332–336. 10.1007/s00246-011-0139-x 22057244

[55] Vanker A, Gie RP, Zar HJ (2017) The association between environmental tobacco smoke exposure and childhood respiratory disease: a review. Expert Rev Respir Med 11: 661–673. 10.1080/17476348.2017.1338949 28580865 PMC6176766

[56] Brook I (2011) The impact of smoking on oral and nasopharyngeal bacterial flora. J Dent Res 90: 704–710. 10.1177/0022034510391794 21558542

[57] Oliver JR, Pierse N, Stefanogiannis N, Jackson C, Baker MG (2017) Acute rheumatic fever and exposure to poor housing conditions in New Zealand: A descriptive study. J Paediatr Child Health 53: 358–364. 10.1111/jpc.13421 28052445

[58] Tadele H, Mekonnen W, Tefera E (2013) Rheumatic mitral stenosis in children: more accelerated course in sub-Saharan patients. BMC Cardiovasc Disord 13: 95 10.1186/1471-2261-13-95 24180350 PMC4228389

[59] Okello E, Wanzhu Z, Musoke C, Twalib A, Kakande B, et al (2013) Cardiovascular complications in newly diagnosed rheumatic heart disease patients at Mulago Hospital, Uganda. Cardiovasc J Afr 24: 80–85. 10.5830/CVJA-2013-004 23736132 PMC3721959

[60] Lubega S, Aliku T, Lwabi P (2014) Echocardiographic pattern and severity of valve dysfunction in children with rheumatic heart disease seen at Uganda Heart Institute, Mulago hospital. Afr Health Sci 14: 617–625. 10.4314/ahs.v14i3.17 25352880 PMC4209653

[61] Badiani S, van Zalen J, Saheecha S, Hart L, Topham A, et al (2017) Clinical events and echocardiographic lesion progression rate in subjects with mild or moderate aortic regurgitation. Echo Res Pract 4: 37–44. 10.1530/ERP-17-0002 28611061 PMC5516543

[62] Bassili A, Zaher RS, Abdel-Fattah M, Tognoni A (2000) Profile of secondary prophylaxis among children with rheumatic heart disease in Alexandria, Egypt. East Mediter Health J 11556035

[63] Guilherme L, Ramasawmy R, Kalil J (2007) Rheumatic fever and rheumatic heart disease: genetics and pathogenesis. Scand J Immunol 66: 199–207. 10.1111/j.1365-3083.2007.01974.x 17635797

[64] Guilherme L, Kalil J (2015) Rheumatic fever: how streptococcal throat infection triggers an autoimmune disease. Infect Immun: Elsevier. pp. 479–493.

[65] Robey WH, Finland M (1930) Effect of tonsillectomy on the acute attack of rheumatic fever: preliminary report. Arch Intern Med 45: 772–782.

[66] Finland M, Robey WH, Heimann H (1933) The effect of tonsillectomy on the occurrence and course of acute polyarthritis: An analysis of 654 consecutive case histories. Amr Heart J 8: 343–356. PMC2194457 21409053

[67] Chamovitz R, Rammelkamp CH, Wannamaker LW, Denny FW (1960) The effect of tonsillectomy on the incidence of streptococcal respiratory disease and its complications. Pediatrics 26: 355–367. 13692248

[68] Anonymous (1970) Tonsillectomy after rheumatic fever. Lancet 1: 708. 4191007

[69] Powell J, O’Hara J, Carrie S, Wilson JA(2017) Is tonsillectomy recommended in adults with recurrent tonsillitis? Br Med J 357: j1450. 28408369 10.1136/bmj.j1450

[70] Gove S, Cardona PN, Tulloch J (1998) Streptococcal pharyngitis in Egyptian children. Lancet 351: 64–65. 10.1016/S0140-6736(05)78058-2 9433444

[71] Sabry NA, Farid SF, Dawoud DM (2014) Antibiotic dispensing in Egyptian community pharmacies: an observational study. Res Social Adm Pharm 10: 168–184. 10.1016/j.sapharm.2013.03.004 23665078

[72] Maraghy DAE, Younis AM, Abbas N (2016) Survey on the irrational use of antibiotics among adults in Egyptian community. Int J Pharmacol Pharm Sci 3: 6–9.

[73] Elboray SN, Little P, Nehal M, Marzouk D, Reda M (2018) Assessment of different clinical variables associated with group a streptococcal throat infection among children in primary care practice. Egypt J Commun Med 36.

[74] Steinhoff MC, Abd el Khalek MK, Khallaf N, Hamza HS, el Ayadi A, et al (1997) Effectiveness of clinical guidelines for the presumptive treatment of streptococcal pharyngitis in Egyptian children. Lancet 350: 918–921. 10.1016/s0140-6736(97)03317-5 9314870

[75] Lue HC, Wu MH, Wang JK, Wu FF, Wu YN (1994) Long-term outcome of patients with rheumatic fever receiving benzathine penicillin G prophylaxis every three weeks versus every four weeks. J Pediatr 125: 812–816. 10.1016/s0022-3476(94)70082-6 7965439

[76] Manyemba J, Mayosi BM (2002) Penicillin for secondary prevention of rheumatic fever. Cochrane Database Syst Rev: CD002227 10.1002/14651858.CD002227 12137650 PMC7017848

[77] Kassem AS, Madkour AA, Massoud BZ, Zaher SR (1992) Benzathine penicillin G for rheumatic fever prophylaxis: 2-weekly versus 4-weekly regimens. Indian J Pediatr 59: 741–748. 10.1007/BF02859412 1340864

[78] Kassem AS, Zaher SR, Abou Shleib H, el-Kholy AG, Madkour AA, et al (1996) Rheumatic fever prophylaxis using benzathine penicillin G (BPG): two- week versus four-week regimens: comparison of two brands of BPG. Pediatrics 97: 992–995. 8637789

[79] Peterson DC (2013) Complications of physician misdiagnosis/treatment of rheumatic fever in the United States. Adv Biosci Biotechnol 4: 143.

[80] Pichichero ME, Disney FA, Talpey WB, Green JL, Francis AB, et al (1987) Adverse and beneficial effects of immediate treatment of Group A beta-hemolytic streptococcal pharyngitis with penicillin. Pediatr Infect Dis J 6: 635–643. 10.1097/00006454-198707000-00004 3302916

[81] el-Daher NT, Hijazi SS, Rawashdeh NM, al-Khalil IA, Abu-Ektaish FM, et al (1991) Immediate vs. delayed treatment of group A beta-hemolytic streptococcal pharyngitis with penicillin V. Pediatr Infect Dis J 10: 126–130. 10.1097/00006454-199102000-00010 1905799

[82] Gerber MA, Randolph MF, DeMeo KK, Kaplan EL (1990) Lack of impact of early antibiotic therapy for streptococcal pharyngitis on recurrence rates. J Pediatr 117: 853–858. 10.1016/s0022-3476(05)80121-0 2123239

[83] Pichichero ME, Casey JR (2007) Systematic review of factors contributing to penicillin treatment failure in Streptococcus pyogenes pharyngitis. Otolaryngol Head Neck Surg 137: 851–857. 10.1016/j.otohns.2007.07.033 18036409

[84] Balbaa A, ElGuindy A, Pericak D, Yacoub MH, Schwalm JD (2015) An evaluation of secondary prophylaxis for rheumatic heart disease in rural Egypt. Glob Cardiol Sci Pract: 40 10.5339/gcsp.2015.40 26779516 PMC4633577

[85] Dale JB, Batzloff MR, Cleary PP, Courtney HS, Good MF, et al (2016) Current approaches to group A streptococcal vaccine development. The University of Oklahoma Health Sciences Center. 2016/02/12 ed. Oklahoma. 26866216

[86] World Health Organization (2018) Vaccines against Strep A. Geneva: WHO.

[87] De Rosa G, Pardeo M, Stabile A, Rigante D (2006) Rheumatic heart disease in children: from clinical assessment to therapeutical management. Eur Rev Med Pharmacol Sci 10: 107–110. 16875042

[88] Williamson L, Bowness P, Mowat A, Ostman-Smith I (2000) Lesson of the week: difficulties in diagnosing acute rheumatic fever-arthritis may be short lived and carditis silent. Br Med J 320: 362–365. 10657336 10.1136/bmj.320.7231.362 PMC1127146

[89] Khriesat I, Najada AH (2003) Acute rheumatic fever without early carditis: an atypical clinical presentation. Eur J Pediatr 162: 868–871. 10.1007/s00431-003-1320-x 14648218

[90] Grossman BJ, Athreya B (1962) Sources of errors in diagnosis of acute rheumatic fever in children. JAMA 182: 830–833. 10.1001/jama.1962.03050470008002 13950825

[91] Marks M, Marks JL (2016) Viral arthritis. Clin Med 16: 129–134. 10.7861/clinmedicine.16-2-129 PMC4868140 27037381

[92] Chen L, Xie X, Gu J, Xu L, Yang X, et al (2009) Changes of manifestations of 122 patients with rheumatic fever in South China during last decade. Rheumatol Int 30: 239–243. 10.1007/s00296-009-0944-1 19444451

[93] Figueroa FE, Fernandez MS, Valdes P, Wilson C, Lanas F, et al (2001) Prospective comparison of clinical and echocardiographic diagnosis of rheumatic carditis: long term follow up of patients with subclinical disease. Heart 85: 407–410. 10.1136/heart.85.4.407 11250966 PMC1729708

[94] Aliku T, Beaton A, Scheel A, Tompsett A, Lwabi P, et al (2015) Evaluating the risk of rheumatic heart disease among family members of children identified with latent RHD in school-based echocardiographic screening. Circulation 132: A19428–A19428.

[95] Bryant PA, Robins-Browne R, Carapetis JR, Curtis N (2009) Some of the people, some of the time: susceptibility to acute rheumatic fever. Circulation 119: 742–753. 10.1161/CIRCULATIONAHA.108.792135 19204317

[96] Gray LA, D'Antoine HA, Tong SYC, McKinnon M, Bessarab D, et al (2017) Genome-wide analysis of genetic risk factors for rheumatic heart disease in aboriginal Australians provides support for pathogenic molecular mimicry. J Infect Dis 216: 1460–1470. 10.1093/infdis/jix497 29029143

[97] Tong SY, D'Antoine H, McKinnon M, Turner K, Hudson M, et al (2020) Lessons learned in genetic research with Indigenous Australian participants. Med J Australia 212: 200–202.e201. 10.5694/mja2.50499 32017112

[98] Stanevicha V, Eglite J, Sochnevs A, Gardovska D, Zavadska D, et al (2003) HLA class II associations with rheumatic heart disease among clinically homogeneous patients in children in Latvia. Arthritis Res Ther 5: R340–346. 10.1186/ar1000 14680508 PMC333411

[99] Stanevicha V, Eglite J, Zavadska D, Sochnevs A, Shantere R, et al (2007) HLA class II DR and DQ genotypes and haplotypes associated with rheumatic fever among a clinically homogeneous patient population of Latvian children. Arthritis Res Ther 9: R58 10.1186/ar2216 17559688 PMC2206337

[100] Muhamed B, Parks T, Sliwa K (2020) Genetics of rheumatic fever and rheumatic heart disease. Nat Rev Cardiol 17: 145–154. 10.1038/s41569-019-0258-2 31519994

[101] El-Hagrassy N, El-Chennawi F, Zaki ME-S, Fawzy H, Zaki A, et al (2010) HLA class I and class II HLA DRB profiles in Egyptian children with rheumatic valvular disease. Pediatric Cardiology 31: 650–656. 10.1007/s00246-010-9663-3 20145915

[102] Chou HT, Chen CH, Chen JY, Chang KC (2008) Association of HLA DRB1-DQA1-DQB1 haplotypes with rheumatic heart disease in Taiwan. Int J Cardiol 128: 434–435. 10.1016/j.ijcard.2007.06.032 17673320

[103] World Medical Association (2013) World Medical Association Declaration of Helsinki. Ethical principles for medical research involving human subjects. Ferney-Voltaire: WMA. 10.1001/jama.2013.281053 24141714

