# Supplementary material for: How accurate is the diagnosis of rheumatic fever in Egypt? Data from the national rheumatic heart disease prevention and control program (2006-2018)
Source: PLoS Negl Trop Dis. 2020 Aug 17;14(8):e0008558. doi: 10.1371/journal.pntd.0008558 (PMC7451991; doi:10.1371/journal.pntd.0008558)
Supplement: S1 Table — (DOCX) [file pntd.0008558.s001.docx]

Table S1: Regimens of Benzathine Penicillin G in primary and secondary prophylaxis of rheumatic fever in Egypt

| Antibiotic | Prophylaxis | Mode of administration | Dose |
| --- | --- | --- | --- |
| BPG | Primary  Secondary | Single IM injection  Single IM injection every 2 weeks (locally manufactured BPG)  every 3 – 4 weeks (Brand BPG) | ≥30kg: 1.2 million IU  <30kg: 600,000 IU |

BPG; Benzathine Penicillin G

IM; Intramuscular
