# Supplementary material for: How accurate is the diagnosis of rheumatic fever in Egypt? Data from the national rheumatic heart disease prevention and control program (2006-2018)
Source: PLoS Negl Trop Dis. 2020 Aug 17;14(8):e0008558. doi: 10.1371/journal.pntd.0008558 (PMC7451991; doi:10.1371/journal.pntd.0008558)
Supplement: S2 Table — (DOCX) [file pntd.0008558.s002.docx]

Table S2: Final diagnosis in relation to recurrent attacks of tonsillitis, tonsillectomy and use of BPG for ARF secondary prophylaxis

|  | | **Diagnosis** | | | | | | | | | | | | | | **Univariate analysis** |
| --- | --- | --- | --- | --- | --- | --- | --- | --- | --- | --- | --- | --- | --- | --- | --- | --- |
|  |  | **Normal~** | | **Misdiagnosis** | | **RHD** | | **Rheumatic arthritis** | | **Sydenham’s chorea** | | **RHD and Sydenham’s chorea** | | **RHD and R. arthritis** | |  |
|  |  | **No.** | **%** | **No.** | **%** | **No.** | **%** | **No.** | **%** | **No.** | **%** | **No.** | **%** | **No.** | **%** | ***p*** |
| Recurrent attacks of tonsillitis | | 2542 | 29.7 | 3261 | 38.0 | 925 | 10.8 | 1650 | 19.2 | 1 | 0.01 | 5 | 0.06 | 188 | 2.2 | **<0.001** |
| Tonsillectomy | | 1572 | 28.1 | 2256 | 40.4 | 583 | 10.4 | 1052 | 18.8 | 1 | 0.02 | 2 | 0.04 | 120 | 2.1 | **<0.001** |
| BPG [Yes] | | 0 | 0.0 | 6411 | 65.2 | 1203 | 12.2 | 1972 | 20.0 | 1 | 0.01 | 2 | 0.02 | 228 | 2.3 | **<0.001** |
| Adherent | | 0 | 0.0 | 3984 | 68.6 | 782 | 13.5 | 923 | 15.9 | 0 | 0.0 | 1 | 0.02 | 105 | 1.8 | **<0.001** |
| Non-adherent | | 0 | 0.0 | 2427 | 60.3 | 421 | 10.5 | 1049 | 26.0 | 1 | 0.02 | 1 | 0.02 | 123 | 3.1 |  |
| BPG Regimen | 2 Weeks | 0 | 0.0 | 5595 | 66.1 | 1023 | 12.1 | 1630 | 19.3 | 1 | 0.01 | 1 | 0.01 | 198 | 2.3 | **<0.001** |
|  | 3 Weeks | 0 | 0.0 | 158 | 62.2 | 31 | 12.2 | 57 | 22.4 | 0 | 0.0 | 1 | 0.40 | 7 | 2.8 |  |
|  | 4 Weeks | 0 | 0.0 | 658 | 58.9 | 149 | 13.3 | 285 | 25.5 | 0 | 0.0 | 0 | 0.00 | 23 | 2.1 |  |

BPG= Benzathine penicillin G

RHD= rheumatic heart disease

~19 patients in this category reported use BPG as of ARF primary prophylaxis
