# Supplementary material for: How accurate is the diagnosis of rheumatic fever in Egypt? Data from the national rheumatic heart disease prevention and control program (2006-2018)
Source: PLoS Negl Trop Dis. 2020 Aug 17;14(8):e0008558. doi: 10.1371/journal.pntd.0008558 (PMC7451991; doi:10.1371/journal.pntd.0008558)
Supplement: S3 Table — (DOCX) [file pntd.0008558.s003.docx]

Table S3: Recurrent attacks of tonsillitis in relation to history of tonsillectomy and use of BPG for ARF secondary prophylaxis

|  | **Recurrent attacks of tonsillitis** | | | | **Univariate analysis** | **Multivariate analysis** | | | |
| --- | --- | --- | --- | --- | --- | --- | --- | --- | --- |
|  | **Yes (n=5807)** | | **No**  **(n=6541)** | |  | **Exp(B)** | **95% C.I. for EXP(B)** | | ***P*** |
|  | **No.** | **%** | **No.** | **%** | ***p*** |  | **LL** | **UL** |  |
| BPG [Yes] | 5391 | 62.9 | 4445 | 52.7 | **<0.001** | **8.70** | **7.20** | **10.50** | **<0.001** |
| Adherent | 2695 | 31.4 | 3114 | 36.9 | **<0.001** | **0.33** | **0.29** | **0.37** | **<0.001** |
| Non-adherent | 2696 | 31.5 | 1331 | 15.8 |  |  |  |  |  |
| BPG Regimen |  |  |  |  | **<0.001** |  |  |  |  |
| 2 Weeks | 4333 | 50.5 | 4131 | 48.9 |  | **0.22** | **0.19** | **0.27** | **<0.001** |
| 3 Weeks | 184 | 2.1 | 70 | 0.8 |  | **0.62** | **0.42** | **0.90** | **0.013** |
| 4 Weeks | 874 | 10.2 | 244 | 2.9 |  |  |  |  |  |
| Tonsillectomy | 5591 | 65.2 | 48 | 0.6 | **<0.001** | **367.1** | **275.0** | **490.0** | **<0.001** |

BPG= benzathine penicillin G

C.I.=confidence interval
