# Supplementary material for: How accurate is the diagnosis of rheumatic fever in Egypt? Data from the national rheumatic heart disease prevention and control program (2006-2018)
Source: PLoS Negl Trop Dis. 2020 Aug 17;14(8):e0008558. doi: 10.1371/journal.pntd.0008558 (PMC7451991; doi:10.1371/journal.pntd.0008558)
Supplement: S4 Table — (DOCX) [file pntd.0008558.s004.docx]

Table S4: Degree of valvular involvement and the associated factors among rheumatic patients

|  | | Degree of valvular affection | | | | | | | | | |  |
| --- | --- | --- | --- | --- | --- | --- | --- | --- | --- | --- | --- | --- |
|  |  | Normal (n=14877) | | Trivial (n=241) | | Mild (n=1635) | | Moderate n=220) | | Sever n=41) | | *p* |
|  |  | No. | % | No. | % | No. | % | No. | % | No. | % |  |
| Familial ARF/RHD | No | 14196 | 95.4 | 235 | 97.5 | 1562 | 95.5 | 215 | 97.3 | 37 | 90.2 | 0.154 |
|  | Yes | 680 | 4.6 | 6 | 2.5 | 73 | 4.5 | 6 | 2.7 | 4 | 9.8 |  |
| Age | <5 | 712 | 4.8 | 5 | 2.1 | 28 | 1.7 | 2 | 0.9 | 2 | 4.9 | <0.001 |
|  | 5 – <15 | 9900 | 66.6 | 141 | 58.5 | 833 | 50.9 | 114 | 51.6 | 7 | 17.1 |  |
|  | 15 – <30 | 3480 | 23.4 | 64 | 26.6 | 479 | 29.3 | 68 | 30.8 | 12 | 29.3 |  |
|  | 30 – 50 | 760 | 5.1 | 30 | 12.4 | 256 | 15.7 | 30 | 13.6 | 13 | 31.7 |  |
|  | 50+ | 24 | 0.2 | 1 | 0.4 | 39 | 2.4 | 7 | 3.2 | 7 | 17.1 |  |
| Smoking | No | 11137 | 74.9 | 131 | 54.4 | 1240 | 75.8 | 141 | 63.8 | 35 | 85.4 | <0.001 |
|  | Yes | 257 | 1.7 | 18 | 7.5 | 102 | 6.2 | 13 | 5.9 | 5 | 12.2 |  |
|  | Passive | 3482 | 23.4 | 92 | 38.2 | 293 | 17.9 | 67 | 30.3 | 1 | 2.4 |  |
| Sex | Male | 5651 | 38.0 | 51 | 21.2 | 486 | 29.7 | 58 | 26.2 | 13 | 31.7 | <0.001 |
|  | Female | 9225 | 62.0 | 190 | 78.8 | 1149 | 70.3 | 163 | 73.8 | 28 | 68.3 |  |
| Residence | Rural | 9165 | 61.6 | 169 | 70.1 | 916 | 56.0 | 143 | 64.7 | 23 | 56.1 | <0.001 |
|  | Urban | 5711 | 38.4 | 72 | 29.9 | 719 | 44.0 | 78 | 35.3 | 18 | 43.9 |  |
| Family size | 1 – 3 | 883 | 5.9 | 18 | 7.5 | 112 | 6.9 | 10 | 4.5 | 3 | 7.3 | 0.001 |
|  | 4 – 8 | 13838 | 93.0 | 223 | 92.5 | 1488 | 91.0 | 206 | 93.2 | 37 | 90.2 |  |
|  | >8 (9 – 15) | 155 | 1.0 | 0 | 0.0 | 35 | 2.1 | 5 | 2.3 | 1 | 2.4 |  |
| Crowding index | 1 – 2 | 14319 | 96.3 | 237 | 98.3 | 1549 | 94.7 | 207 | 93.7 | 39 | 95.1 | 0.026 |
|  | 3 – 5 | 492 | 3.3 | 3 | 1.2 | 74 | 4.5 | 13 | 5.9 | 2 | 4.9 |  |
|  | >5 (6 – 11) | 65 | 0.4 | 1 | 0.4 | 12 | 0.7 | 1 | 0.5 | 0 | 0.0 |  |
| Household Ventilation | Good | 10380 | 69.8 | 234 | 97.1 | 1031 | 63.1 | 157 | 71.0 | 24 | 58.5 | <0.001 |
|  | Average | 4082 | 27.4 | 6 | 2.5 | 535 | 32.7 | 52 | 23.5 | 15 | 36.6 |  |
|  | Bad | 414 | 2.8 | 1 | 0.4 | 69 | 4.2 | 12 | 5.4 | 2 | 4.9 |  |
| Recurrent attacks of tonsillitis | None | 7422 | 49.9 | 186 | 77.2 | 710 | 43.4 | 103 | 46.6 | 21 | 51.2 | <0.001 |
|  | Yes | 7454 | 50.1 | 55 | 22.8 | 925 | 56.6 | 118 | 53.4 | 20 | 48.8 |  |
|  | ≤6 per year | 6493 | 43.6 | 55 | 22.8 | 814 | 49.7 | 98 | 44.3 | 16 | 39.0 |  |
|  | > 6 per year | 961 | 6.5 | 0 | 0.0 | 111 | 6.9 | 20 | 9.0 | 4 | 9.8 |  |
| Tonsillectomy | No | 9995 | 67.2 | 187 | 77.6 | 1073 | 65.6 | 139 | 62.9 | 34 | 82.9 | <0.001 |
|  | Yes | 4881 | 32.8 | 54 | 22.4 | 562 | 34.4 | 82 | 37.1 | 7 | 17.1 |  |
| Hospitalization | No | 13713 | 92.2 | 237 | 98.3 | 1453 | 88.9 | 185 | 83.7 | 29 | 70.7 | <0.001 |
|  | ARF/RHD | 109 | 0.7 | 1 | 0.4 | 24 | 1.5 | 11 | 5.0 | 9 | 22.0 |  |
|  | Others | 1054 | 7.1 | 3 | 1.2 | 158 | 9.7 | 25 | 11.3 | 3 | 7.3 |  |
| BPG | No | 6473 | 43.5 | 114 | 47.3 | 525 | 32.1 | 58 | 26.2 | 8 | 19.5 | <0.001 |
|  | Yes | 8403 | 56.5 | 127 | 52.7 | 1110 | 67.9 | 163 | 73.8 | 33 | 80.5 |  |
| Adherence | Adherent | 4921 | 33.1 | 119 | 49.4 | 662 | 40.5 | 86 | 38.9 | 21 | 51.2 | <0.001 |
|  | Non-adherent | 3482 | 23.4 | 8 | 3.3 | 448 | 27.4 | 77 | 34.8 | 12 | 29.3 |  |
| BPG Regimen | 2 Weeks | 7242 | 48.7 | 126 | 52.3 | 927 | 56.7 | 138 | 62.4 | 31 | 75.6 | <0.001 |
|  | 3 Weeks | 215 | 1.4 | 0 | 0.0 | 32 | 2.0 | 6 | 2.7 | 1 | 2.4 |  |
|  | 4 Weeks | 946 | 6.4 | 1 | 0.4 | 151 | 9.2 | 19 | 8.6 | 1 | 2.4 |  |

ARF= acute rheumatic fever

BPG= benzathine penicillin G

RHD= rheumatic heart disease
