# Supplementary material for: How accurate is the diagnosis of rheumatic fever in Egypt? Data from the national rheumatic heart disease prevention and control program (2006-2018)
Source: PLoS Negl Trop Dis. 2020 Aug 17;14(8):e0008558. doi: 10.1371/journal.pntd.0008558 (PMC7451991; doi:10.1371/journal.pntd.0008558)
Supplement: S5 Table — (DOCX) [file pntd.0008558.s005.docx]

Table S5: Factors associated with of rheumatic fever and its sequelae among family members of RHD patients who had valve replacement surgery

|  | **Screened children/relatives**  **of RHD patients (n=769)** | | | | **Univariate analysis** | **Multivariate analysis** | | | |
| --- | --- | --- | --- | --- | --- | --- | --- | --- | --- |
|  | **Rheumatic (n=159)** | | **Normal**  **(n=610)** | |  | **Exp(B)** | **95% C.I. for EXP(B)** | | ***p*** |
|  | **n** | **%** | **n** | **%** | *p* |  | **LL** | **UL** |  |
| Age (Years) | | | | | | | | | |
| <5 | 4 | 2.5 | 52 | 8.5 | **<0.001** |  |  |  |  |
| 5 – <15 | 93 | 58.5 | 453 | 74.3 |  |  |  |  |  |
| 15 – <30 | 58 | 36.5 | 94 | 15.4 |  |  |  |  |  |
| 30 – 50 | 4 | 2.5 | 11 | 1.8 |  |  |  |  |  |
| **(Mean ± SD)** | 13.2 ± 6.5 | | 10.3 ± 5.7 | | **<0.001** |  |  |  |  |
| **Smoking** | | | | | |  |  |  |  |
| Yes | 0 | 0.0 | 1 | 0.2 | ND |  |  |  |  |
| passive | 18 | 11.3 | 79 | 13.0 | 0.577 |  |  |  |  |
| **Sex** |  |  |  |  |  |  |  |  |  |
| Male | 65 | 40.9 | 314 | 51.5 | **0.018** |  |  |  |  |
| Female | 94 | 59.1 | 296 | 48.5 |  |  |  |  |  |
| **Residence** |  |  |  |  |  |  |  |  |  |
| Rural | 78 | 49.1 | 318 | 52.1 | **<0.001** |  |  |  |  |
| Urban | 81 | 50.9 | 292 | 47.9 |  |  |  |  |  |
| **Education** |  |  |  |  |  |  |  |  |  |
| Illiterate | 10 | 6.3 | 124 | 20.3 | **<0.001** |  |  |  |  |
| Read and Write | 5 | 3.1 | 7 | 1.1 |  |  |  |  |  |
| Kindergarten | 5 | 3.1 | 18 | 3.0 |  |  |  |  |  |
| Primary | 69 | 43.4 | 310 | 50.8 |  |  |  |  |  |
| Preparatory | 33 | 20.8 | 80 | 13.1 |  |  |  |  |  |
| Secondary | 31 | 19.5 | 54 | 8.9 |  |  |  |  |  |
| University | 613 | 5.0 | 297 | 6.4 |  |  |  |  |  |
| **Father's occupation** |  |  |  |  |  |  |  |  |  |
| Working | 3 | 1.9 | 26 | 4.3 |  |  |  |  |  |
| Not working | 139 | 87.4 | 548 | 89.8 | **0.001** |  |  |  |  |
| Retired | 7 | 4.4 | 7 | 1.1 |  |  |  |  |  |
| **Mother's occupation** |  |  |  |  |  |  |  |  |  |
| Working | 137 | 86.2 | 526 | 86.2 |  |  |  |  |  |
| Not Working | 20 | 12.6 | 80 | 13.1 | 0.379 |  |  |  |  |
| **Parents** |  |  |  |  |  |  |  |  |  |
| Alive | 150 | 94.3 | 599 | 98.2 | 0.309 |  |  |  |  |
| Died | 9 | 5.6 | 11 | 1.8 |  |  |  |  |  |
| **Marital status** |  |  |  |  |  |  |  |  |  |
| Single | 24 | 61.5 | 49 | 64.5 | 0.757 |  |  |  |  |
| Married | 15 | 38.5 | 27 | 35.5 |  |  |  |  |  |
| **Income** |  |  |  |  |  |  |  |  |  |
| Not enough | 93 | 58.5 | 389 | 63.8 | 0.221 |  |  |  |  |
| Enough | 66 | 41.5 | 221 | 36.2 |  |  |  |  |  |
| **Family Size** |  |  |  |  |  |  |  |  |  |
| 1 – 3 | 6 | 3.8 | 40 | 6.6 | 0 .242 |  |  |  |  |
| 4 – 8 | 152 | 95.6 | 567 | 93.0 |  |  |  |  |  |
| >8 (9 – 15) | 1 | 0.6 | 3 | 0.5 |  |  |  |  |  |
| **(Mean ± SD)** | 5.0 ± 1.2 | | 4.7 ± 1.0 | | **0.002** | **1.30** | **1.07** | **1.57** | **0.008** |
| **Crowding Index** |  |  |  |  |  |  |  |  |  |
| 1 – 2 | 151 | 95.0 | 592 | 97.0 |  |  |  |  |  |
| 3 – 5 | 4 | 2.5 | 16 | 2.6 | 0.185 | 0.76 | 0.18 | 3.17 | 0.711 |
| >5 (6 – 11) | 4 | 2.5 | 2 | 0.3 |  | **8.57** | **1.01** | **72.86** | **0.049** |
| **Ventilation** |  |  |  |  |  |  |  |  |  |
| Good | 97 | 61.0 | 428 | 70.2 |  |  |  |  |  |
| Average | 59 | 37.1 | 172 | 28.2 | **0.046** |  |  |  |  |
| Bad | 3 | 1.9 | 10 | 1.6 |  |  |  |  |  |
| **Medical service** |  |  |  |  |  |  |  |  |  |
| None | 50 | 31.4 | 146 | 23.9 | **<0.001** | **3.33** | **1.17** | **9.45** | **0.024** |
| Private health care services | 73 | 45.9 | 236 | 38.7 |  | 1.77 | 0.64 | 4.88 | 0.269 |
| Health Insurance | 26 | 16.4 | 172 | 28.2 |  | 1.20 | 0.41 | 3.52 | 0.744 |
| PHC units | 4 | 2.5 | 14 | 2.3 |  | 3.20 | 0.58 | 17.6 | 0.182 |
| University Hospitals | 0 | 0.0 | 2 | 0.3 |  | 0.00 | 0.00 | 0.00 | 0.999 |
| Public Hospitals | 6 | 3.8 | 40 | 6.6 |  |  |  |  | **0.017** |
| **Referral method** |  |  |  |  |  |  |  |  |  |
| Health care Physician | 154 | 96.9 | 459 | 75.2 | **<0.001** | **3.79** | **1.01** | **14.13** | **0.048** |
| Family | 1 | 0.6 | 47 | 7.7 |  | 0.41 | 0.04 | 4.50 | 0.467 |
| Advertisement | 1 | 0.6 | 59 | 9.7 |  | 0.24 | 0.02 | 2.63 | 0.241 |
| Friend | 0 | 0.0 | 2 | 0.3 |  | 0.00 | 0.00 | 0.00 | 1.000 |
| Others | 3 | 1.9 | 43 | 7.0 |  |  |  |  | **<0.001** |
| **Symptoms** |  |  |  |  |  |  |  |  |  |
| Tonsillitis | 106 | 66.7 | 331 | 54.3 | **0.005** |  |  |  |  |
| Pharyngitis | 20 | 12.6 | 44 | 7.2 | **0.031** | **2.04** | **1.05** | **3.98** | **0.040** |
| Breathlessness/Dyspnea on effort | 67 | 42.1 | 213 | 34.9 | 0.093 | 1.49 | 0.95 | 2.33 | 0.080 |
| Arthralgia | 1 | 0.6 | 21 | 3.4 | 0.092 |  |  |  |  |
| Arthritis | 60 | 37.7 | 30 | 4.9 | **<0.001** | **11.43** | **6.62** | **19.75** | **<0.001** |
| Fever | 4 | 2.5 | 13 | 2.1 | 0.769 |  |  |  |  |
| Others | 0 | 0.0 | 11 | 1.8 | ND |  |  |  |  |
| **Recurrent attacks of tonsillitis** | | | | | |  |  |  |  |
| Yes | 105 | 66.0 | 326 | 53.4 | **< 0.001** |  |  |  |  |
| ≤ 6 attacks per year | 85 | 81.0 | 294 | 90.2 | **0.005** |  |  |  |  |
| > 6 attacks per year (up to 27) | 20 | 19.0 | 32 | 9.8 |  |  |  |  |  |
| **Tonsillectomy** | | | | | |  |  |  |  |
| Yes | 57 | 35.8 | 188 | 30.8 | 0.557 |  |  |  |  |
| **Hospitalization** | | | | | |  |  |  |  |
| Cardiac | 4 | 2.5 | 0 | 0.0 | **0.007** |  |  |  |  |
| Others | 17 | 10.7 | 60 | 9.8 |  |  |  |  |  |
| **Echocardiography** | | | | | |  |  |  |  |
| Normal | 70 | 44.0 | 610 | 100.0 |  |  |  |  |  |
| MS | 4 | 2.5 | 0 | 0.0 |  |  |  |  |  |
| MR | 73 | 45.9 | 0 | 0.0 |  |  |  |  |  |
| AR | 4 | 2.5 | 0 | 0.0 |  |  |  |  |  |
| TR | 5 | 3.1 | 0 | 0.0 |  |  |  |  |  |
| MS + AR | 3 | 1.9 | 0 | 0.0 |  |  |  |  |  |
| **Degree of valvular affection** |  |  |  |  |  |  |  |  |  |
| Trivial | 6 | 3.8 | 0 | 0.0 |  |  |  |  |  |
| Mild | 73 | 45.9 | 0 | 0.0 |  |  |  |  |  |
| Moderate | 6 | 3.8 | 0 | 0.0 |  |  |  |  |  |
| Sever | 4 | 2.5 | 0 | 0.0 |  |  |  |  |  |
| **Diagnosis** |  |  |  |  |  |  |  |  |  |
| Normal | 0 | 0.0 | 556 | 91.1 |  |  |  |  |  |
| Others (misdiagnosis) | 76 | 47.8 | 0 | 0.0 |  |  |  |  |  |
| RHD | 70 | 44.0 | 0 | 0.0 |  |  |  |  |  |
| Rheumatic arthritis | 0 | 0.0 | 54 | 8.9 |  |  |  |  |  |
| RHD and Sydenham’s chorea | 13 | 8.2 | 0 | 0.0 |  |  |  |  |  |
| **Major criteria of Rheumatic fever** |  |  |  |  |  |  |  |  |  |
| Carditis | 89 | 56.0 | 0 | 0.0 |  |  |  |  |  |
| Arthritis | 83 | 52.2 | 0 | 0.0 |  |  |  |  |  |
| **History of taking BPG** |  |  |  |  |  |  |  |  |  |
| Yes | 85 | 53.5 | 61 | 10.0 | **<0.001** |  |  |  |  |
| **BPG regimen** |  |  |  |  |  |  |  |  |  |
| 2 weeks | 72 | 84.7 | 49 | 80.3 | **<0.001** |  |  |  |  |
| 3 weeks | 1 | 1.2 | 3 | 4.9 |  |  |  |  |  |
| 4 weeks | 12 | 14.1 | 9 | 14.8 |  |  |  |  |  |
| **BPG Frequency** |  |  |  |  |  |  |  |  |  |
| Adherent | 39 | 45.9 | 36 | 59.0 | **<0.001** |  |  |  |  |
| Non-adherent | 46 | 54.1 | 25 | 41.0 |  |  |  |  |  |
| Misdiagnosis | 0 | 0.0 | 54 | 8.9 | **0.039** |  |  |  |  |

AR= Aortic regurgitation; AS= Aortic stenosis; ASOT= Antistreptolysin-O titre; BPG= benzathine penicillin G; C.I.=confidence interval; ESR= erythrocyte sedimentation rate; MR= mitral regurgitation; MS= mitral stenosis; PHC= primary health care; RHD= rheumatic heart disease; TR= tricuspid regurgitation
