# Supplementary figures and images for: How accurate is the diagnosis of rheumatic fever in Egypt? Data from the national rheumatic heart disease prevention and control program (2006-2018)
Source: PLoS Negl Trop Dis. 2020 Aug 17;14(8):e0008558. doi: 10.1371/journal.pntd.0008558 (PMC7451991; doi:10.1371/journal.pntd.0008558)

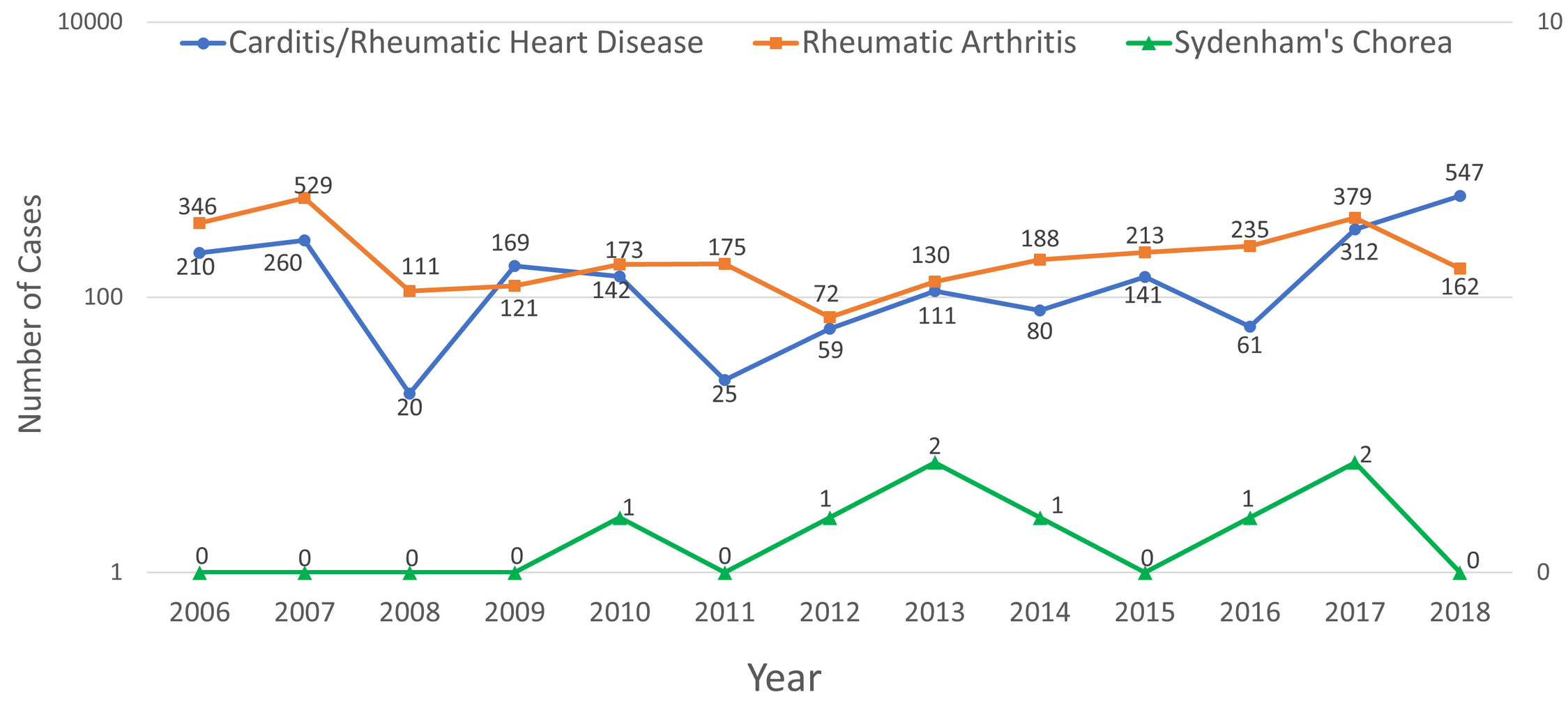

Supplement: S1 Fig — (TIF) [file pntd.0008558.s006.tif]

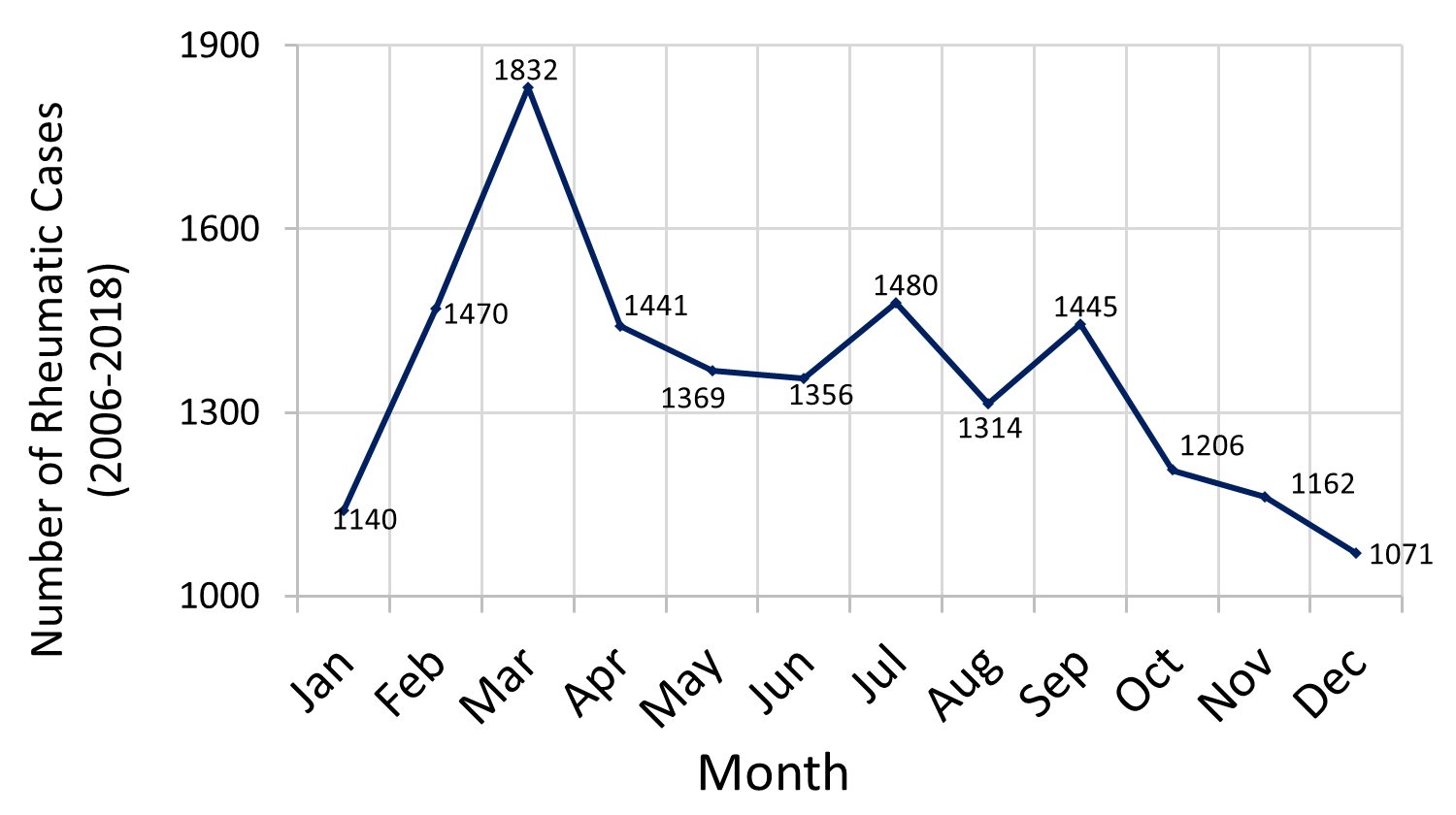

Supplement: S2 Fig — (TIF) [file pntd.0008558.s007.tif]
